# A review of global environmental mercury processes in response to human and natural perturbations: Changes of emissions, climate, and land use

**DOI:** 10.1007/s13280-017-1004-9

**Published:** 2018-01-31

**Authors:** Daniel Obrist, Jane L. Kirk, Lei Zhang, Elsie M. Sunderland, Martin Jiskra, Noelle E. Selin

**Affiliations:** 1 Department of Environmental, Earth and Atmospheric Sciences, University of Massachusetts, Lowell, One University Ave, Lowell, MA 01854 USA; 2 Environment and Climate Change, Canada, 867 Lakeshore Road, Burlington, ON L7P 2X3 Canada; 3 School of the Environment, Nanjing University, 163 Xianlin Avenue, Nanjing, 210023 Jiangsu China; 4 Harvard John A. Paulson School of Engineering and Applied Sciences, Harvard T.H. Chan School of Public Health, Harvard University, 29 Oxford Street, Cambridge, MA 02138 USA; 5 Present Address: Environmental Geosciences, University of Basel, Bernoullistr. 30, 4056 Basel, Switzerland; 6 Géosciences Environnement Toulouse, GET-CNRS, CNRS – OMP, 14 Avenue Edouard Belin, 31400 Toulouse, France; 7 Institute for Data, Systems, and Society and Department of Earth, Atmospheric and Planetary Sciences, Massachusetts Institute of Technology, 77 Massachusetts Avenue, Cambridge, MA 02139 USA

**Keywords:** Climate change, Emission change, Heavy metal, Land use change, Mercury global environmental cycling

## Abstract

We review recent progress in our understanding of the global cycling of mercury (Hg), including best estimates of Hg concentrations and pool sizes in major environmental compartments and exchange processes within and between these reservoirs. Recent advances include the availability of new global datasets covering areas of the world where environmental Hg data were previously lacking; integration of these data into global and regional models is continually improving estimates of global Hg cycling. New analytical techniques, such as Hg stable isotope characterization, provide novel constraints of sources and transformation processes. The major global Hg reservoirs that are, and continue to be, affected by anthropogenic activities include the atmosphere (4.4–5.3 Gt), terrestrial environments (particularly soils: 250–1000 Gg), and aquatic ecosystems (e.g., oceans: 270–450 Gg). Declines in anthropogenic Hg emissions between 1990 and 2010 have led to declines in atmospheric Hg^0^ concentrations and Hg^II^ wet deposition in Europe and the US (− 1.5 to − 2.2% per year). Smaller atmospheric Hg^0^ declines (− 0.2% per year) have been reported in high northern latitudes, but not in the southern hemisphere, while increasing atmospheric Hg loads are still reported in East Asia. New observations and updated models now suggest high concentrations of oxidized Hg^II^ in the tropical and subtropical free troposphere where deep convection can scavenge these Hg^II^ reservoirs. As a result, up to 50% of total global wet Hg^II^ deposition has been predicted to occur to tropical oceans. Ocean Hg^0^ evasion is a large source of present-day atmospheric Hg (approximately 2900 Mg/year; range 1900–4200 Mg/year). Enhanced seawater Hg^0^ levels suggest enhanced Hg^0^ ocean evasion in the intertropical convergence zone, which may be linked to high Hg^II^ deposition. Estimates of gaseous Hg^0^ emissions to the atmosphere over land, long considered a critical Hg source, have been revised downward, and most terrestrial environments now are considered net sinks of atmospheric Hg due to substantial Hg uptake by plants. Litterfall deposition by plants is now estimated at 1020–1230 Mg/year globally. Stable isotope analysis and direct flux measurements provide evidence that in many ecosystems Hg^0^ deposition via plant inputs dominates, accounting for 57–94% of Hg in soils. Of global aquatic Hg releases, around 50% are estimated to occur in China and India, where Hg drains into the West Pacific and North Indian Oceans. A first inventory of global freshwater Hg suggests that inland freshwater Hg releases may be dominated by artisanal and small-scale gold mining (ASGM; approximately 880 Mg/year), industrial and wastewater releases (220 Mg/year), and terrestrial mobilization (170–300 Mg/year). For pelagic ocean regions, the dominant source of Hg is atmospheric deposition; an exception is the Arctic Ocean, where riverine and coastal erosion is likely the dominant source. Ocean water Hg concentrations in the North Atlantic appear to have declined during the last several decades but have increased since the mid-1980s in the Pacific due to enhanced atmospheric deposition from the Asian continent. Finally, we provide examples of ongoing and anticipated changes in Hg cycling due to emission, climate, and land use changes. It is anticipated that future emissions changes will be strongly dependent on ASGM, as well as energy use scenarios and technology requirements implemented under the Minamata Convention. We predict that land use and climate change impacts on Hg cycling will be large and inherently linked to changes in ecosystem function and global atmospheric and ocean circulations. Our ability to predict multiple and simultaneous changes in future Hg global cycling and human exposure is rapidly developing but requires further enhancement.

## Introduction

**Fig. 1:**
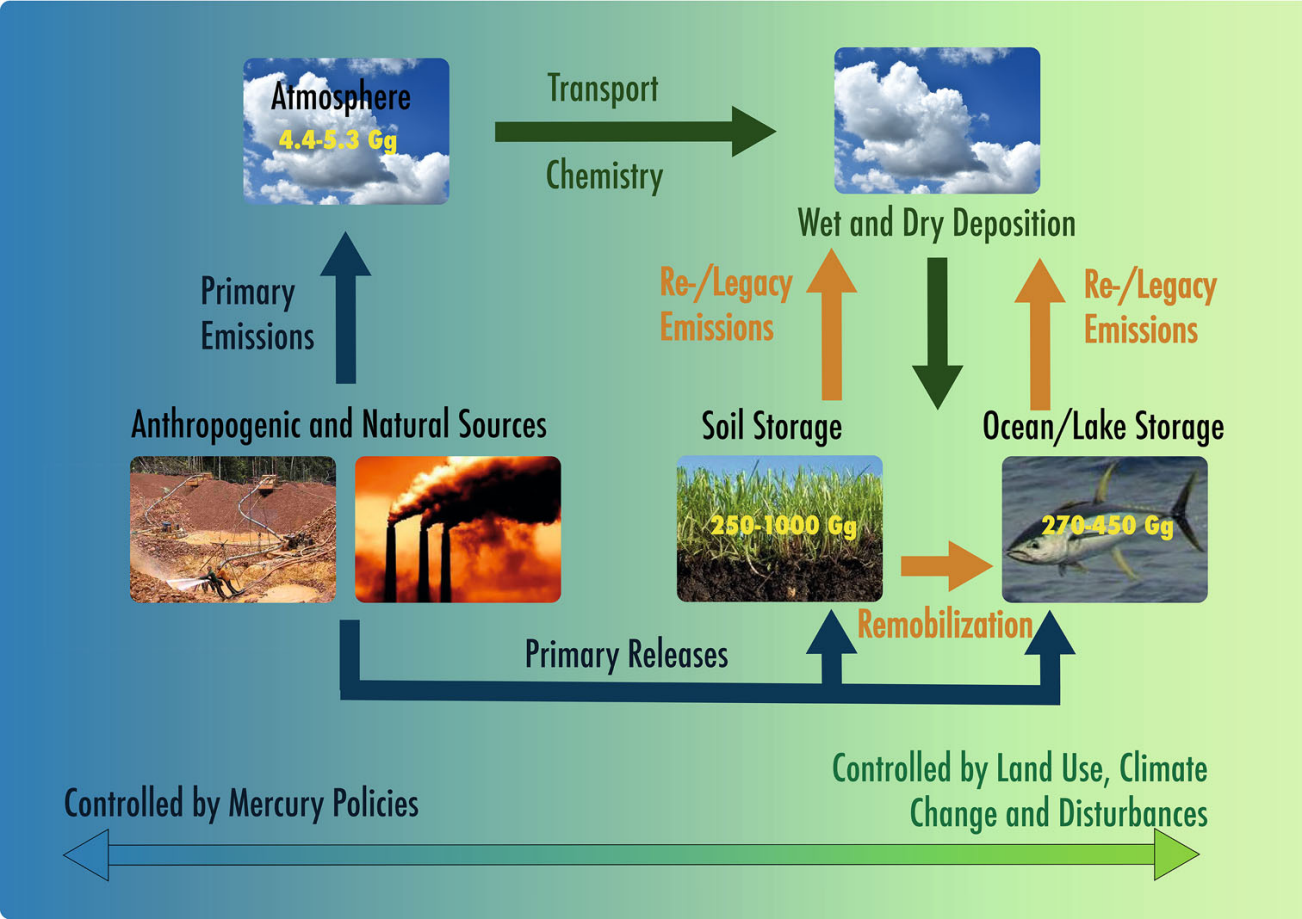
Overview of global Hg cycling and impacts of policies and global change. Yellow numbers are estimated pool sizes in global reservoirs, and arrows indicate exchange processes between major environmental reservoirs. Pool sizes and exchanges are strongly modulated by anthropogenic emissions and releases (blue arrows) which transform in the atmosphere and deposit to aquatic and terrestrial ecosystems (green arrows). The Hg cycle will continue to experience disturbances due to changes in anthropogenic emissions and will be increasingly affected by land use and climate change, which remobilize (orange arrows) Hg that has accumulated in environmental reservoirs from previous Hg emissions and releases

Our understanding of the critical processes driving global mercury (Hg) cycling, in particular those that affect large-scale exchange of Hg among major environmental compartments, has advanced substantially over the past decade. Progress has been driven by major advances in three interconnected areas: new data, new models, and new analytical tools and techniques. In this paper, we summarize the state of knowledge of the major global Hg reservoirs in the Earth system: the atmosphere, terrestrial ecosystems, and aquatic ecosystems. We describe the constraints on processes that control Hg exchanges between these reservoirs, and the relative influences of policy, land use, climate change, and anthropogenic disturbances on Hg cycling (Fig. 1).

Analyses of newly available data in the context of advances in modeling capabilities and novel analysis techniques have improved our understanding of fundamental processes relevant to Hg cycling. In the past decade, new data have become available from areas of the world where they previously were lacking, including Asia, the tropics, and the southern hemisphere. Environmental models are increasingly used for synthesizing global observations and describing the mechanisms driving Hg speciation, cycling, and bioavailability. Global three-dimensional (3D) models of Hg in the atmosphere (Dastoor and Larocque 2004; Selin et al. 2007; Jung et al. 2009; Travnikov and Ilyin 2009; Holmes et al. 2010; Durnford et al. 2012; Bieser et al. 2017; Horowitz et al. 2017), terrestrial ecosystems (Smith-Downey et al. 2010), and oceans (Zhang et al. 2014, 2015b; Semeniuk and Dastoor 2017) have improved our understanding of Hg processes. A major advance has been the development of a hierarchy of modeling tools that collapse the necessary detail from global simulations into more computationally feasible geochemical box models, enabling fully coupled simulations of the interactions among the land, atmosphere, and oceans over millennial time scales (Amos et al. 2013, 2014, 2015). When combined with information on the cumulative history of human Hg release from antiquity to the present, this modeling approach has revealed a much greater contribution of human activity to the global Hg cycle than previously recognized (Streets et al. 2011, 2017). The last 10 years has also seen rapid development in Hg stable isotope biogeochemistry, providing a valuable tool to quantify Hg sources and study transformation processes. Incorporation of Hg stable isotopes in global models has the potential to constrain the relative importance of specific sources and processes (Sonke 2011; Sun et al. 2016a) (See Box 1).

**Box 1:** Use of stable isotopes to constrain Hg sources and processes

The seven stable isotopes of Hg (196, 198, 199, 200, 201, 202, and 204) are subject to different mass-dependent (MDF) and mass-independent (MIF) isotope fractionation mechanisms. The isotopic signature of Hg in a sample carries information on its initial source and the transformation processes it underwent. Here we provide a simplified three-dimensional view of Hg stable isotope systematics and focus on exemplary processes and sources that have pronounced identifiable effects on Hg isotope signatures in environmental samples. For a more comprehensive introduction to Hg isotope fractionation and a compilation of Hg isotope variation in natural samples, we refer to the reviews of Blum et al. (2014) and Blum and Johnson (2017).	
*MDF (mass-dependent fractionation)* is reported as δ^202^Hg in the “small delta” notation, representing the ^202/198^Hg ratio of a sample relative to the NIST SRM3133 international standard as defined by Blum and Bergquist (2007). Primary natural and anthropogenic Hg emission sources are generally associated with small negative δ^202^Hg signatures, whereas atmospheric Hg pools have positive δ^202^Hg values (Blum et al. 2014; Sun et al. 2016b). All biotic and abiotic transformation processes enrich Hg stable isotopes to different extents by MDF. One of the largest MDF fractionations observed is associated with preferential uptake of light atmospheric Hg^0^ by foliage (arrow 1 in Fig. 3) (Demers et al. 2013; Enrico et al. 2016; Yu et al. 2016).	
*Odd-MIF (mass-independent fractionation of odd-mass-number Hg isotopes)* is reported as Δ^199^Hg and Δ^201^Hg in the “capital delta” notation, representing the anomalies of the ^199/198^Hg and ^201/198^Hg ratios from the MDF line, respectively (Blum and Bergquist 2007). Two independent mechanisms have been experimentally shown to cause odd-MIF: (1) magnetic isotope effects (MIE) (Buchachenko 2001, 2009) and (2) nuclear volume fractionation (NVF) (Bigeleisen 1996; Schauble 2007). Primary Hg sources are generally associated with circum-zero odd-MIF (Sun et al. 2016b). Largest odd-MIF signatures are associated with MIE by photochemical reactions, whereas odd-MIF associated with NVF (identifiable by a different Δ^199^Hg/Δ^201^Hg ratio) is relatively small. Photochemical demethylation of MeHg to Hg^0^ has been shown to cause large odd-MIF, leading to an enrichment of odd-mass Hg isotopes in the residual MeHg that is preserved along the aquatic food chain (arrow 2 in Fig. 3) (Bergquist and Blum 2007). Photoreduction of Hg^II^ complexes in snow was shown to lead to large depletions of odd-mass isotopes in residual snow (arrow 3 in Fig. 3) (Sherman et al. 2010).	
*Even-MIF (mass-independent fractionation of even-mass-number Hg isotopes)* is reported as Δ^200^Hg and Δ^204^Hg, representing anomalies of the ^200/198^Hg and ^204/198^Hg ratios from the MDF line (Blum and Bergquist 2007). The potential mechanisms causing even-MIF have not been identified yet, but are possibly related to oxidation of Hg^0^ by halogen radicals in the atmosphere (Sun et al. 2016a). Hg^II^ in precipitation, representing atmospheric gaseous and aerosol Hg^II^, is consistently enriched in positive anomalies of Δ^200^Hg (and concurrent negative Δ^204^Hg) (Gratz et al. 2010; Chen et al. 2012; Sherman et al. 2012; Demers et al. 2013; Enrico et al. 2016). The complementary pool of atmospheric Hg^0^ is enriched in negative Δ^200^Hg (and concurrent positive Δ^204^Hg) anomalies (Gratz et al. 2010; Demers et al. 2013, 2015, Enrico et al. 2016; Obrist et al. 2017). Note that the possible mechanisms for even-MIF are not necessarily associated with the ^200^Hg and ^204^Hg isotopes only and may also affect the ^202/198^Hg ratio that is used for normalization.	

These recent advances have proven particularly valuable for investigating the anticipated impacts of human and natural perturbations on global Hg cycling. Changes in anthropogenic emissions are ongoing and will continue into the future, including strong shifts in global source areas compared to current emission patterns (Giang et al. 2015). Accelerating land use and climate change are expected to have significant effects on global, regional, and local Hg cycles, with unexpected feedbacks and nonlinear impacts on Hg exposure. Models have been applied to assess the impact of regulatory interventions, such as emission controls (Selin et al. 2018), on specific outcomes and to evaluate policy efforts to mitigate Hg pollution (Selin 2014). An increasing number of studies are now available documenting such changes.

Here, we review the major insights gained from scientific advances over the past decade on global Hg cycling and Hg exchanges within and among the environmental compartments of the atmosphere, terrestrial ecosystems, and aquatic ecosystems. We then synthesize this knowledge to assess the impacts of human activities, including those affected by Hg-specific and other environmental policies, on the future of global Hg cycling. Further detailed discussion of factors affecting aquatic Hg loading, Hg methylation and demethylation processes, and exposure of wildlife and humans to Hg in the context of environmental change and disturbances are provided by Eagles-Smith et al. (2018) and Hsu-Kim et al. (2018), while relevant scientific insights for global policy are described in Selin et al. (2018).

## Recent advances in understanding critical Hg cycling processes of global importance

**Fig. 2:**
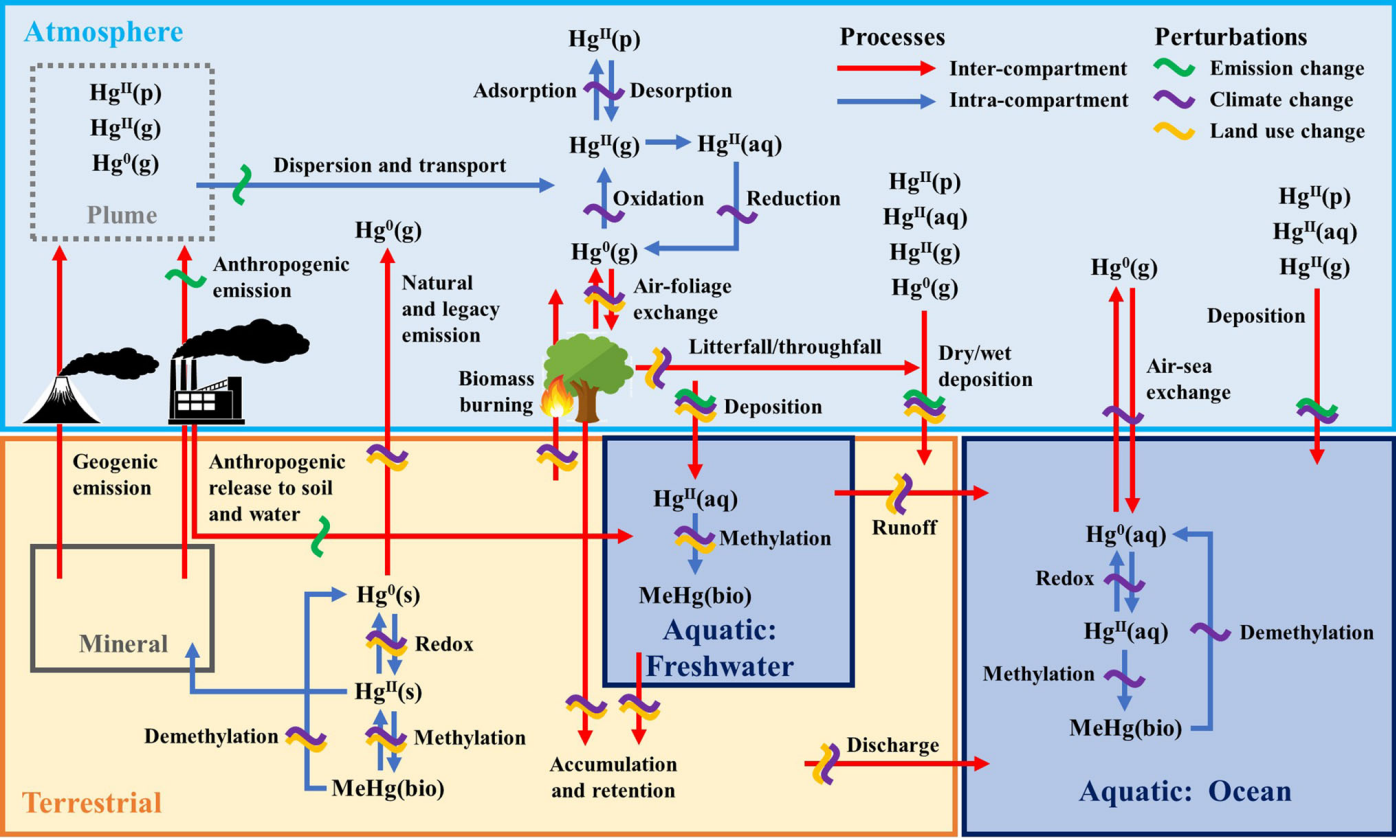
Critical processes of global importance for Hg cycling, including fluxes between major environmental compartments. Perturbations of Hg processes and fluxes show anticipated impacts due to changes in emission, climate, and land use. A detailed discussion of the relevant processes is found in the “Recent advances in understanding critical Hg cycling processes of global importance” section, and major disturbances to global Hg cycling are discussed in the “Anticipated impacts from human and natural perturbations, including emission changes and climate change, on global Hg cycling” section

In this section, we discuss critical processes of importance for Hg cycling within (Fig. 2, blue arrows) and between (Fig. 2, red arrows) major environmental compartments (atmosphere, terrestrial, and aquatic [freshwater and ocean]).

### Atmosphere

Atmospheric total gaseous mercury (TGM) concentrations have been measured since the late 1970s, with more reliable observations emerging since approximately 1990 (Slemr et al. 2003; Lindberg et al. 2007; Temme et al. 2007; Sprovieri et al. 2010; Slemr et al. 2011; Sprovieri et al. 2016). Mean TGM concentrations in background areas in the northern hemisphere, the tropics, and the southern hemisphere are 1.3–1.6, 1.1–1.3, and 0.8–1.1 ng/m^3^, respectively (Slemr et al. 2011; Sprovieri et al. 2016). Many observations show that in the past three decades global background TGM concentrations have declined, particularly in North America, Europe, and over the North Atlantic Ocean (Slemr et al. 2003; Lindberg et al. 2007; Temme et al. 2007; Slemr et al. 2008; Ebinghaus et al. 2011; Slemr et al. 2011; Soerensen et al. 2012; Cole et al. 2013; Weigelt et al. 2015; Sprovieri et al. 2016), with rates of declines between − 1.5 and − 2.2% per year. Because anthropogenic emission inventories did not support such declines, proposed drivers for declines include decreased Hg concentrations and subsequent evasion in the upper North Atlantic Ocean and/or changes in terrestrial surface–atmosphere fluxes (Slemr et al. 2011; Soerensen et al. 2012). Others have shown that declining point-source emissions of divalent Hg (Hg^II^) in the northern hemisphere and from commercial products (Horowitz et al. 2014) were sufficient to reproduce observed declining trends in TGM from 1995 to present (Zhang et al. 2016d). TGM concentrations have decreased at lower rates in parts of the Arctic (approximately 0 to − 0.2% per year) (Cole et al. 2013). Several studies have suggested that the recent reversal in global TGM trends in China and India are due to increasing emissions from coal-fired power plants in addition to artisanal and small-scale gold mining (ASGM) activity (Slemr et al. 2014; Martin et al. 2017; Streets et al. 2017). At Cape Point in South Africa, TGM has increased during the last decade, although a clear explanation for this trend is lacking (Martin et al. 2017).

The global atmospheric Hg reservoir is estimated to be between 4400 and 5300 Mg, and is enriched by more than an order of magnitude relative to natural levels and by approximately three- to fivefold relative to 1850 levels (Amos et al. 2013; Engstrom et al. 2014; Horowitz et al. 2017; Streets et al. 2017). The anthropogenic contribution to atmospheric Hg has been inferred from observed Hg deposition in polar firn air, peat bogs, and lake sediments, all of which reveal large-scale impacts of anthropogenic emissions, particularly in the second half of the twentieth century (Biester et al. 2007; Faïn et al. 2009; Farmer et al. 2009; Outridge et al. 2011; Goodsite et al. 2013; Engstrom et al. 2014; Amos et al. 2015; Blais et al. 2015; Drevnick et al. 2016). Combining deposition records with Hg stable isotope analysis now allows differentiation between atmospheric Hg^II^ and Hg^0^ deposition, and reconstruction of past Hg^0^ levels (Enrico et al. 2016, 2017). Enrico et al. (2017) suggest that the maximum twentieth-century atmospheric Hg^0^ concentration was 15 times larger than natural background at various locations, which agrees well with modeling analyses by Amos et al. (2015). Reconstructions suggest that historical atmospheric Hg^0^ concentrations peaked around 1970 at 3–4 ng/m^3^ and then declined to the current levels of 1.3–1.6 ng/m^3^ (Faïn et al. 2009; Enrico et al. 2017), consistent with atmospheric Hg^0^ concentration measurements of the last few decades. Lake sediment archives, reflecting total atmospheric Hg deposition, show similar time trends, with maximum Hg accumulation rates around 1970–1990 (Engstrom et al. 2014; Amos et al. 2015; Drevnick et al. 2016).

Direct anthropogenic Hg emissions account for approximately 30% of total annual inputs (i.e., total anthropogenic and natural emissions plus re-emission) to the atmosphere (Pirrone et al. 2010; UNEP 2013; Streets et al. 2017). While several different emission inventories exist for the present day, few are comparable across the past decades (Muntean et al. 2014) or address all-time human emissions (Streets et al. 2011; Horowitz et al. 2014; Streets et al. 2017). Global anthropogenic Hg emissions have been estimated at approximately 2000 Mg/year by most studies (AMAP/UNEP 2008, 2013; Pacyna et al. 2010; Pirrone et al. 2010; Streets et al. 2011) except for one that reported a lower estimate (EDGARv4) of 1287 Mg/year (Muntean et al. 2014). While reference years of studies are within a common period of 2005–2010, the sectoral distributions among some studies are inconsistent. The Arctic Monitoring and Assessment Programme and United Nations Environment Programme (AMAP/UNEP) (2008) and studies by Pacyna et al. (2010), Pirrone et al. (2010), and Streets et al. (2011) found stationary fossil fuel combustion (SFFC), mainly coal combustion, to be the leading source of Hg (800–900 Mg/year), followed by ASGM (400 Mg/year). By assuming a higher activity level of unregulated and illegal ASGM in some countries, UNEP’s (2013) global Hg assessment (AMAP/UNEP 2013) estimated ASGM emissions of more than 700 Mg/year. SFFC accounted for less than 500 Mg/year of Hg in the UNEP inventory, while the EDGARv4 inventory (Muntean et al. 2014) estimated lower contributions from SFFC and ASGM. Given the global importance of ASGM releases, this source warrants further constraints. National-level emission inventories rely on local field emission tests, with an increasing amount of data emerging from newly industrialized countries. For example, Zhang et al. (2015a) estimated anthropogenic Hg emissions in China to be 538 Mg for 2010, somewhat lower than a previous estimate of 609 Mg/year (Pirrone et al. 2010). The discrepancy was attributed to better Hg removal efficiencies of air pollution control devices for nonferrous metal smelting (Wang et al. 2010; Zhang et al. 2012). Emissions in India were recently estimated at approximately 240 Mg/year (Pirrone et al. 2010; Burger Chakraborty et al. 2013). National anthropogenic Hg emission estimates are available for several other countries (e.g., Kim et al. 2010; Masekoameng et al. 2010; Nansai et al. 2012; Nelson et al. 2012; US EPA 2015). As per the UNEP emission inventory, the eight highest emitting countries are (in order) China, India, Indonesia, Columbia, South Africa, Russia, Ghana, and the U.S. These countries emit a total of 1095 Mg/year of Hg, or 56% of the global anthropogenic Hg emissions, to the atmosphere (AMAP/UNEP 2013; UNEP 2013). More accurate emission inventories are expected in the future, as parties of the UNEP Minamata Convention on Hg improve national Hg use and emission inventories.

Uncertainties remain in global anthropogenic Hg emission inventories, mainly because of a lack of local field test data for key sectors such as cement production, iron and steel production, waste incineration, and gold production, and a lack of accurate activity-level data for ASGM and intentional Hg use. Horowitz et al. (2014) found that more than 400 Mg/year of atmospheric Hg emission was from the intentional use of Hg in products and processes (excluding gold production), previously unaccounted for in inventories. Top-down constraints and inverse modeling from atmospheric observations are currently being explored as additional tools to better constrain emissions (Song et al. 2015, 2016; Denzler et al. 2017).

Hg speciation profiles of emissions are crucial to assess the environmental impacts of atmospheric emissions (Zhang et al. 2016b). Streets et al. (2005) reported overall Hg^0^:Hg_gas_^II^:Hg_particulate_^II^ ratios of 20:78:2 and 80:15:5 for Chinese coal-fired power plants (CFPPs) and cement plants (CPs), respectively, based on North American and European field test data. A recent study by Zhang et al. (2015a) updated the speciation profiles to 79:21:0 and 34:65:1 for Chinese CFPPs and CPs, respectively, based on onsite measurements. Such changes in speciation profiles for large point sources lead to large variations in Hg transport distances and local deposition fluxes.

Hg^0^ oxidation to Hg^II^ is considered a key step in removing Hg from the atmosphere (Selin et al. 2008; Lyman and Jaffe 2011; Subir et al. 2011), although increasing evidence shows that oxidized Hg^II^ deposition is dominant only in oceans, while Hg^0^ deposition is dominant in many terrestrial ecosystems (see below). A recent controversy has arisen around the techniques and artifacts in measuring atmospheric Hg oxidation and atmospheric Hg^II^ concentrations. We refer to published reviews on atmospheric Hg and Hg^II^ measurement methodologies (Lyman et al. 2010b; McClure et al. 2014a, b; Jaffe et al. 2014; Gustin et al. 2015; Angot et al. 2016; Mao et al. 2016; Marusczak et al. 2017). Earlier work suggested a primary role for the oxidants OH and O_3_ in Hg^0^ oxidation, but observations and theoretical calculations now suggest that the Br radical is likely the dominant oxidant (Hynes et al. 2009; Gratz et al. 2015; Shah et al. 2016). Current thinking is that the mechanism is a two-step process, where Hg^0^ reacts with the Br radical to form HgBr, and HgBr reacts with multiple potential oxidants (Br, I, OH, BrO, IO, NO_2_, etc.) to generate different Hg^II^ species (Goodsite et al. 2004; Dibble et al. 2012; Wang et al. 2014; Ye et al. 2016; Horowitz et al. 2017; Jiao and Dibble 2017). Recent aircraft observations showed substantial atmospheric Hg^II^ concentrations and detectable BrO in the subtropics, suggesting that subtropical anticyclones are significant global Hg^II^ sources (Gratz et al. 2015; Shah et al. 2016). Photolytic formation of halogen radicals and Hg oxidation may occur at low light conditions, and recent Antarctic wintertime Hg oxidation may suggest a possible dark oxidation process (Nerentorp Mastromonaco et al. 2016). Hg oxidation chemistry has recently been updated in the GEOS-Chem model by Horowitz et al. (2017), which describes in-cloud Hg^II^ photoreduction as potentially important in Hg redox chemistry (Holmes et al. 2010; Horowitz et al. 2017). A recent model comparison (Travnikov et al. 2017), not including the updated chemistry of Horowitz et al., showed that models with a Br oxidation mechanism reproduced the observed near-surface seasonal variations in the Hg^II^ fraction, but did not capture seasonal variations in wet deposition observed at monitoring sites in North America and Europe. These findings suggest that more complex Hg oxidation chemistry and multi-oxidation pathways may be occurring in different regions of the atmosphere.

### Terrestrial

In the terrestrial environment, the largest Hg pools are located in soils (Grigal 2003; Obrist 2012). Using global soil carbon inventories, Hg pools have been estimated at 300 Gg (Hararuk et al. 2013) and 240 Gg (Smith-Downey et al. 2010). The latter study estimated a 20% modern-day increase in overall soil storage and an even greater increase of Hg associated with the labile soil carbon fraction (Smith-Downey et al. 2010). Newer simulations using additional observational constraints (Streets et al. 2011; Horowitz et al. 2014) estimate higher present-day organic soil Hg pools (250–1000 Gg with a best estimate of 500 Gg) and propose that anthropogenic activities have doubled the Hg stored in organic soils (Amos et al. 2013, 2015). Hg in organic-rich upper soils is predominantly from atmospheric deposition (Grigal et al. 2000; Schwesig and Matzner 2000; Guedron et al. 2006; Peña-Rodríguez et al. 2012; Demers et al. 2013; Peña-Rodríguez et al. 2014; Jiskra et al. 2015; Enrico et al. 2016; Zheng et al. 2016; Obrist et al. 2017; Wang et al. 2017). Atmospheric Hg deposits are retained in humus-rich upper soils and bound to organic matter (Meili 1991; Grigal 2003; Obrist et al. 2011; Jiskra et al. 2015). In mineral horizons, a significant component of Hg also stems from release from natural geologic sources.

New spatial soil datasets demonstrate that landscape Hg distribution is correlated to soil organic matter, latitude, annual precipitation, leaf area index, and vegetation greenness (Obrist et al. 2011; Richardson et al. 2013; Navrátil et al. 2014, 2016; Obrist et al. 2016). Obrist et al. (2016) explained soil Hg variability across 1911 sites in the western U.S., largely by vegetation patterns, with high soil Hg accumulation in productive forests and 2.5 times lower soil concentrations in unproductive deserts and scrublands. Such patterns are due to a dominance of plant-funneled atmospheric Hg^0^ deposition in many terrestrial ecosystems (see below), or by higher re-emission of Hg from deserts compared to forests (Eckley et al. 2016). Soil Hg accumulation and retention, however, are also determined by soil morphology and genesis as well as soil properties, including soil organic matter stability, content, texture, and pH (Obrist et al. 2011; Richardson and Friedland 2015; Navrátil et al. 2016). Landscape gradients with the highest inorganic Hg concentrations in forested watersheds have also been reported in river and lake sediments (Fleck et al. 2016), highlighting strong connectivity between upland Hg deposition and aquatic loading. This is consistent with earlier studies that linked high biological Hg hotspots in the eastern U.S. to watersheds with high forest densities (Driscoll et al. 2007).

Elevation gradients in Hg, largely associated with shifts in vegetation type, have also been observed, with vegetation and organic soil Hg concentrations increasing by up to fourfold with increasing elevation (Zhang et al. 2013; Townsend et al. 2014; Blackwell and Driscoll 2015; Wang et al. 2017). Blackwell and Driscoll (2015) reported a shift in deposition patterns along an alpine forest gradient, from deposition dominated by litterfall (i.e., uptake of Hg by plant foliage and transfer to ecosystems after senescence/leaf shedding) to throughfall deposition (i.e., Hg uptake to plant surfaces and wash-off by precipitation) and deposition via cloudwater. They also noted that cloudwater accounted for up to 71% of total deposition at the highest altitudes. Hg stable isotope studies are inconsistent, reporting both higher contributions of Hg^II^ at high-elevation sites (Zhang et al. 2013; Zheng et al. 2016) and lower contributions of Hg^II^ with increasing altitude (Wang et al. 2017).

### Aquatic

Oceans contain a substantial fraction of the global Hg reservoir and strongly affect atmospheric concentrations through air–sea exchange (Soerensen et al. 2010; Zhang et al. 2015b). Sunderland and Mason (2007) estimated the total ocean Hg to be 350 Gg with a 90% confidence limit of 270–450 Gg. More recent work (Lamborg et al. 2014; Zhang et al. 2015b) suggests a reservoir of 260–280 Gg. Lamborg et al. (2014) estimated the upper ocean (top 1000 m) reservoir, based on observations from the GEOTRACES cruise series, to be 63 Mg, which is on the low end of the range reported by Sunderland and Mason (2007) (63–120 Mg; 90% confidence interval), and may indicate either declining concentrations in some basins or improvements in analytical techniques (Lamborg et al. 2012). In the pelagic marine environment, trends in total Hg (THg) concentration are difficult to infer because of large inter-laboratory variability in measurements (Lamborg et al. 2012). Concentrations in the North Atlantic appear to be declining during the last several decades, from mean vertical profile concentrations of above 5 pM in the 1990s to consistently below 1 pM on recent cruises (Bowman et al. 2016). In the North Pacific, however, Sunderland et al. (2009) suggest that the THg concentration in the North Pacific Intermediate Water (NPIW) mass has increased since the mid-1980s due to enhanced atmospheric deposition from the Asian continent. Other cruises that sampled different locations (Hammerschmidt and Bowman 2012; Munson et al. 2015) did not see statistically different THg concentrations than those measured on earlier cruises, but current data coverage is sparse.

In freshwater ecosystems, predominant Hg sources include direct release from Hg-containing effluents, river runoff that contains atmospheric Hg deposits that accumulated in terrestrial environments, and direct atmospheric Hg deposition. The latter is particularly important in lakes with large surface area-to-volume ratios and small catchment-to-lake surface areas. The relative importance of atmospheric and watershed Hg sources varies [see the “Atmosphere–aquatic interactions” section and Hsu-Kim et al. (2018)] depending on the degree of development and land use change, hydrology, and dissolved organic carbon (DOC) content and composition (Engstrom and Swain 1997; Knightes et al. 2009; Lepak et al. 2015). Kocman et al. (2017) recently developed a first inventory of Hg inputs to freshwater ecosystems and estimated that 800–2200 Mg/year of Hg enters freshwater ecosystems. This is lower than the amount of Hg entering coastal ecosystems estimated by Amos et al. (2014) (5500 ± 2700 Mg/year), in part because it does not include natural mobilization from terrestrial ecosystems. Earlier estimates of global riverine Hg release to the ocean were between 1000 and 2000 Mg/year (Cossa et al. 1997; Sunderland and Mason 2007). ASGM is the largest direct Hg source globally to both land and water (approximately 880 Mg/year); although the proportion released to aquatic ecosystems is not certain, it is estimated to be 50% (Kocman et al. 2017). Streets et al. (2017) suggest that approximately 40% of combined Hg releases to land and water globally are sequestered at the release site rather than traveling in rivers to the ocean. In India and China, industrial Hg sources (from the chlor-alkali industry, Hg and large-scale gold production, nonferrous metal production, and Hg-containing waste) release approximately 86 Mg/year to lakes and rivers, accounting for 51% of global aquatic releases from these sources (Kocman et al. 2017).

Methylmercury (MeHg) production is a critical process that occurs within aquatic ecosystems. The largest source of MeHg to freshwater lakes and wetlands is in situ microbial production, with surface sediments producing larger amounts than the water column. However, the relative contributions of water column versus sediment productions depend on specific characteristics of lakes such as stratification, depth of the anoxic hypolimnion, and organic carbon content of sediments. The activity of methylating microbes is controlled by temperature, redox conditions, pH, and the presence of suitable electron donors (e.g., organic carbon) and acceptors (e.g., sulfate, Fe^III^, methane). The primary controls on inorganic Hg bioavailability include DOC, sulfur, and Hg^II^ concentrations and speciation (Benoit et al. 1999; Boening 2000; Ullrich et al. 2001; Gilmour et al. 2013; Hsu-Kim et al. 2013). A breakthrough paper by Parks et al. (2013) identified a two-gene cluster (*hgcA* and *hgcB*) in microbes involved in Hg methylation. The *hgcA* gene encodes a putative corrinoid protein capable of transferring a methyl group to Hg^II^, and the HgcB protein returns HgcA to a redox state that enables it to receive a new methyl group. Subsequent work identified these genes in many organisms, including sulfate and iron-reducing bacteria and methanogens (Gilmour et al. 2013; Yu et al. 2013; Podar et al. 2015). Several studies have developed *hgcA* probes to screen aquatic sediments for Hg-methylating microorganisms (Bae et al. 2014; Schaefer et al. 2014; Bravo et al. 2015; Du et al. 2017) and to demonstrate that different methylating microbes inhabit various niches, such as regions of wetlands with varying sulfate concentrations (Bae et al. 2014; Schaefer et al. 2014). Recent development of a broad range of probes spanning all known *hgcAB* genes (Christensen et al. 2016) may allow future quantification of the methylating potential in environmental samples.

One conundrum that remains is how to explain high levels of MeHg in marine seawater where MeHg production is still poorly understood. Gionfriddo et al. (2016) found that the micro-aerophilic, nitrite-oxidizing bacterium *Nitrospina*, present in Antarctic sea ice and brine, contains genes that are slight rearrangements of the *hgcAB* genes, suggesting that diverse microbial communities may be capable of methylation. Fitzgerald et al. (2007) suggested that most marine MeHg is produced in ocean margin sediments, but a variety of studies across most major ocean basins have since produced strong evidence for water column MeHg production (Cossa et al. 2009; Sunderland et al. 2009; Heimbürger et al. 2010; Cossa et al. 2011; Lehnherr et al. 2011; Blum et al. 2013). In the last several years, the abundance of ocean water concentration measurements has increased dramatically with new data from the North Pacific (Kim et al. 2017); North Atlantic (Bowman et al. 2014); Equatorial, South Atlantic, and Pacific (Munson et al. 2015; Bowman et al. 2016); Arctic (Heimburger et al. 2015); and Antarctic Oceans (Gionfriddo et al. 2016). In the coastal marine environment, Schartup et al. (2015) used enriched Hg isotope incubation to detect active Hg methylation for the first time in oxic estuarine seawater. Methylation was facilitated by the presence of labile terrestrial DOC and a shift in ionic strength and microbial activity that accompanies the transition into saline waters in estuaries. Similarly, Ortiz et al. (2015) measured active methylation in laboratory experiments with marine snow aggregates.

**Fig. 3:**
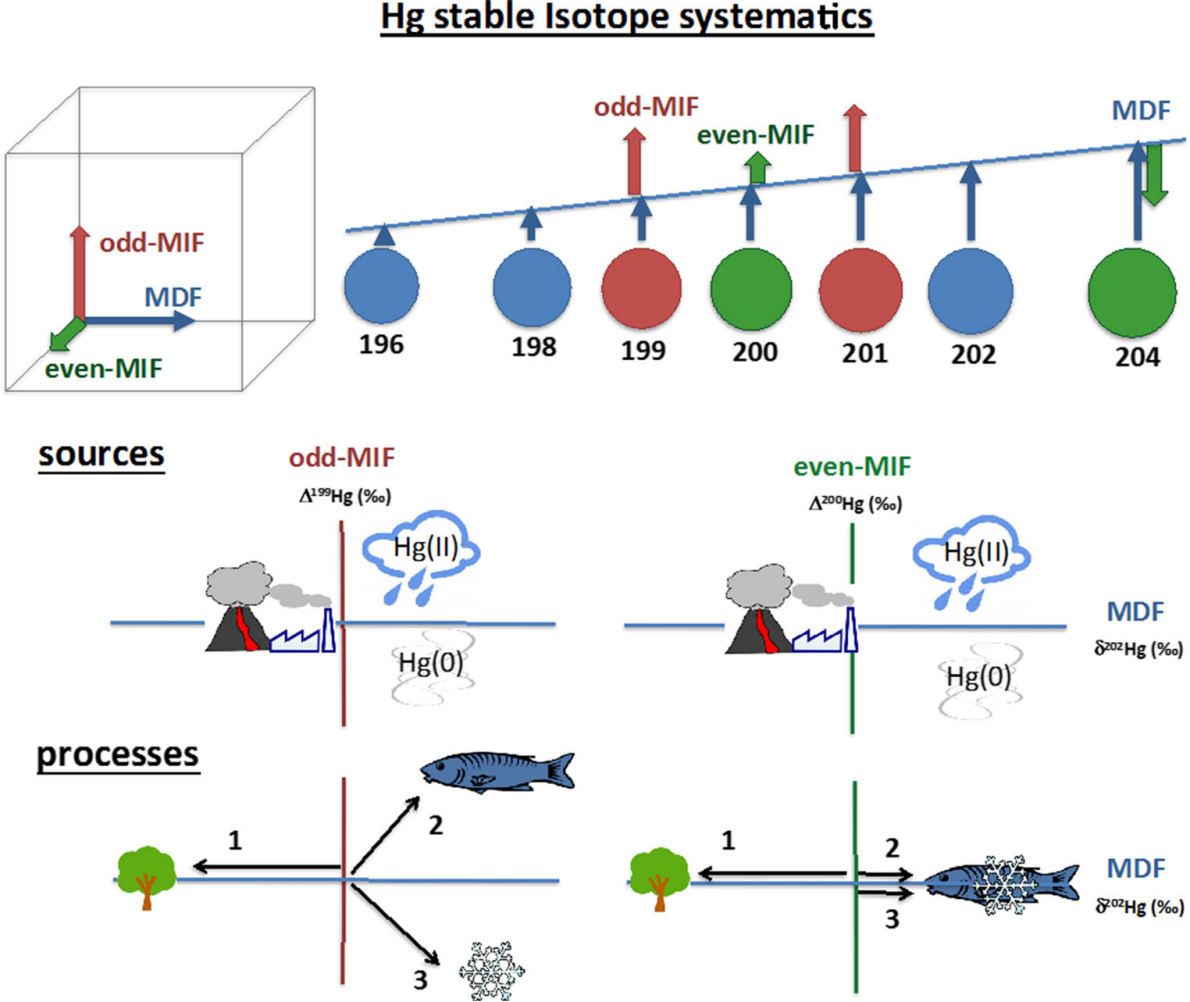
Simplified schematic of Hg stable isotope systematics adapted from Wiederhold et al. (2010). The seven Hg stable isotopes undergo mass-dependent fractionation (MDF) proportional to their atomic mass. Anomalies from the MDF line are defined as mass-independent fractionation (MIF) of the odd-mass-number ^199^Hg and ^201^Hg (odd-MIF) and even-mass-number ^200^Hg and ^204^Hg (even-MIF) isotopes. MDF, odd-MIF, and even-MIF signatures serve as 3D tracers for fingerprinting different Hg sources of natural and anthropogenic emissions, or atmospheric oxidized (Hg^II^) and elemental (Hg^0^) pools. Examples of processes with large isotope fractionation factors in the environment include (1) foliar uptake of Hg^0^ from the atmosphere (Demers et al. 2013; Enrico et al. 2016; Yu et al. 2016); (2) photochemical demethylation of MeHg in waters (Bergquist and Blum 2007); and (3) photochemical reduction of Hg^II^ to Hg^0^ in snow (Sherman et al. 2010)

Marine species are responsible for a large fraction of human exposure to MeHg; for example, Sunderland (2007) found that for the U.S. more than 90% of MeHg exposure is from marine and estuarine species. Thus, understanding MeHg dynamics in marine environments is particularly important for public health (Gribble et al. 2016). Advances have been made in understanding MeHg uptake to aquatic food webs (see Eagles-Smith et al. 2018). The photochemical demethylation of MeHg has been shown experimentally to result in a large enrichment of odd-mass-numbered Hg isotopes (Δ^199^Hg; Fig. 3, Box 1) in the residual MeHg pool that accumulated through the food web (Bergquist and Blum 2007). Odd-mass-independent fractionation (MIF) anomalies in aquatic organisms were used as source tracers to distinguish between MeHg derived from sediments with small odd-MIF isotopes and MeHg from the open ocean with large odd-MIF isotopes (Senn et al. 2010; Blum et al. 2013). Odd-MIF anomalies in biota have been hypothesized to serve as source tracers and as a proxy for ecological parameters such as foraging depth; for example, the largest odd-MIF anomalies are found in fish feeding in surface waters where photochemical demethylation is most active (Blum et al. 2013). The extent of odd-MIF anomaly is reduced in aquatic organisms where light penetration in the aquatic water column is inhibited [e.g., by sea ice (Point et al. 2011; Masbou et al. 2015)] or with high DOC (Sherman and Blum 2013). Thus, odd-MIF anomalies can track changes in climatological/environmental parameters such as sea-ice cover. Field measurements and experimental data suggest that DOC controls MeHg availability for uptake in freshwater. French et al. (2014) observed a water column DOC threshold of 8.6–8.8 mg/L, above which MeHg uptake was inhibited in Arctic lakes, while Jonsson et al. (2014) showed that in estuarine microcosms DOC-bound MeHg was 5–250 times more available for bioaccumulation into lower food web organisms than newly produced MeHg.

### Atmosphere–terrestrial interactions

Large uncertainties in terrestrial Hg^0^ sinks and source strengths, particularly over background areas and vegetated areas, make atmosphere–terrestrial interactions one of least constrained processes in the global Hg cycle. Major emissions from terrestrial ecosystems include biomass burning and volatilization of Hg^0^ from natural and Hg-enriched soils, while deposition from the atmosphere includes dry Hg^0^ deposition to plants and soils and wet Hg^II^ deposition.

Recent global atmospheric models have reported global biomass burning emissions of Hg between 210 and 680 Mg/year (Amos et al. 2014; De Simone et al. 2015; Song et al. 2016). Webster et al. (2016) recently provided an update on atmospheric Hg emissions from western U.S. wildfires and suggest a fivefold lower total (3.1 ± 1.9 Mg/year across 11 states) compared to previous estimates (14.7 Mg/year) (Wiedinmyer and Friedli 2007). The lower Hg emission estimates from Webster et al. are attributed mainly to the inclusion of fire severity and the notion that low-severity burns (52% of wildfires) result in lower soil heating and less Hg emissions. Other studies also found that Hg losses are related to fire intensity (Mitchell et al. 2012; Kolka et al. 2017). Wildfire studies consistently show that fires mobilize Hg primarily from biomass, litter, and organic soils (Friedli et al. 2001, 2003; Engle et al. 2006; Biswas et al. 2007; Obrist 2007; Wiedinmyer and Friedli 2007; Biswas et al. 2008; Burke et al. 2010; Woodruff and Cannon 2010), although Hg released from upper mineral soils can comprise up to 10% of total emissions (Biswas et al. 2007; Burke et al. 2010; Melendez-Perez et al. 2014). Significant post-fire soil evasion of Hg^0^ has been reported to occur years after fires, leading to an additional 50% in Hg emissions (Melendez-Perez et al. 2014), an effect which is not included in wildfire emission estimates.

A relatively well-constrained component of the atmosphere–terrestrial exchange is wet deposition of Hg^II^, at least over land where extensive monitoring networks exist, including the Mercury Deposition Network (MDN) for the U.S. and Canada (Prestbo and Gay 2009; Weiss-Penzias et al. 2016; Sprovieri et al. 2017), the European Monitoring and Evaluation Programme (EMEP) for Europe (Tørseth et al. 2012), and a new Asia–Pacific Mercury Monitoring Network (2017) and the Global Mercury Observation System (GMOS) for other parts of the world. At background sites, mean atmospheric Hg wet deposition globally decreased from 10–15 μg/m^2^/year in the early 1990s to the current level of 5–10 μg/m^2^/year (Zhang et al. 2016d). Declines in atmospheric Hg^II^ wet deposition are reported from Europe and the U.S. with average deposition declines of − 1.5% per year and − 2.3% per year between 1990 and 2013, respectively (Zhang et al. 2016d), although increasing wet Hg^II^ deposition has been reported in East Asia. Mean background Hg wet deposition fluxes in the northern hemisphere, the tropics, and the southern hemisphere are approximately 5–10, 2–8, and 1–5 μg/m^2^/year, respectively (Prestbo and Gay 2009; Fu et al. 2016a; Sprovieri et al. 2016, 2017). There is, however, large spatial and temporal variability in wet Hg^II^ deposition (Butler et al. 2008; Prestbo and Gay 2009; Risch et al. 2012b; Zhang and Jaeglé 2013; Cole et al. 2014; Muntean et al. 2014; Sprovieri et al. 2016).

Estimated median dry deposition fluxes of Hg_gaseous_^II^ and Hg_particulate_^II^ in Asia and North America are 10.7 and 6.1 μg/m^2^/year, respectively. A particular challenge with dry Hg^II^ deposition is that measurements and modeling schemes have uncertainty factors greater than two (Wright et al. 2016), in addition to large measurement uncertainties (see the “Atmosphere” section). Atmospheric dry deposition flux measurements can be broadly divided into micrometeorological methods based on gradient measurements, dynamic gas flux chambers, and surrogate surface passive samplers, which have been more widely used in recent years (Lyman et al. 2009; Zhang et al. 2009; Lyman et al. 2010a; Gustin et al. 2011; Pirrone et al. 2013; Gustin et al. 2015; Huang and Gustin 2015). To model deposition, resistance approaches are used in chemical transport models that apply dry deposition velocities for Hg^II^ and measured or estimated Hg^II^ concentrations (Lin et al. 2006; Zhang et al. 2009). For simulations of net Hg^0^ deposition, bidirectional soil–vegetation–air exchange models are needed (Bash et al. 2007; Zhang et al. 2009); current models are not well constrained. Models have been used to estimate total Hg deposition compared with existing measurements and to attribute deposition to sources in the context of policy. Different models show similar global Hg deposition patterns, especially near source regions, and can reproduce major features of observed wet deposition (Travnikov et al. 2017). However, in some regions, there are substantial differences in simulated attributions of Hg deposition to local versus international sources.

Atmospheric Hg^0^ emissions from land surfaces was previously estimated between 1600 and 2900 Mg/year (Lindberg et al. 2007; Selin et al. 2008; Smith-Downey et al. 2010), but more recent estimates are considerably lower at 607 Mg/year (− 513 to 1353 Mg/year) (Agnan et al. 2016). Estimates of large terrestrial Hg^0^ evasion, or remobilization of legacy Hg from previous depositions, are in part based on enriched isotope tracing studies that showed that 45–70% of applied labile HgCl_2_ was subject to atmospheric evasion (Hintelmann et al. 2002; Graydon et al. 2012). However, recent global data assimilations of 20+ years of terrestrial Hg^0^ flux measurements (Agnan et al. 2016; Zhu et al. 2016) now show that in most of the world Hg^0^ evasion from background soils is generally low, particularly when soils are covered by litter or plants. In fact, many background soils also show periods of net Hg^0^ deposition (Gustin et al. 2000; Choi and Holsen 2009) with recent work showing net Hg^0^ deposition in 20% of measurements over bare soils and in 45% over covered soils (Agnan et al. 2016). On the other hand, Agnan et al. (2016) and Zhu et al. (2016) showed an increased importance of soil Hg^0^ emissions in areas with high atmospheric Hg exposures (such as East Asia), which according to Agnan et al. (2016) now accounts for 261 Mg/year (range of 114–359 Mg/year) of Hg^0^ emissions. They also estimated Hg^0^ evasions from naturally Hg-enriched and contaminated areas (217 Mg/year, range of 202–258 Mg/year) that were lower than previous estimates (Ericksen et al. 2006) but still the second largest terrestrial Hg^0^ source. Another terrestrial Hg^0^ source includes global croplands (201 Mg/year; range 195–208 Mg/year). When considering vegetation uptake of atmospheric Hg, most terrestrial ecosystems turn into net atmospheric Hg^0^ sinks (see below). Recent global model simulations now reflect reduced soil Hg^0^ evasion (Amos et al. 2014, 2015); one global atmospheric model simulation (Song et al. 2016) showed reduced global soil evasions from 2200 to 1360 Mg/year, which is still in the higher range based on flux measurements by Agnan et al. (2016).

Over land, Hg^II^ deposition is minor compared to Hg^0^ deposition, and the dominant pathway of atmospheric deposition to terrestrial ecosystems is through litterfall. Globally, this deposition pathway is estimated at 1020–1230 Mg (Obrist 2007; Risch et al. 2012a; Fu et al. 2016a, b; Wang et al. 2016; Wright et al. 2016; Zhang et al. 2016c). In addition, throughfall deposition may contribute an additional 50% of litterfall deposition (Wang et al. 2016), which would result in a global deposition flux of approximately 560 Mg/year, although this needs further observational confirmation. In addition, further plant depositions occur via woody tissues, including by tree blowdown (Mitchell et al. 2012). Given that Hg contained in woody biomass can exceed Hg contained in foliage biomass by 67–100% (Obrist et al. 2012; Yang et al. 2017), woody tissue deposition is likely an important, yet not well-constrained, source of deposition as well (Obrist 2007). Plant contributions (litterfall plus throughfall wet deposition) dominate deposition loads in forests globally (70–85%), of which approximately 75% (with the exception of South American rainforests) is litterfall (Wang et al. 2016). Wright et al. (2016) and Wang et al. (2016) show large continental differences with high litterfall fluxes in the Amazon and in China that are greater than fivefold those in North America and Europe. This suggests that deposition fluxes decrease spatially from tropical to temperate/boreal regions, with 30% of total deposition in the temperate and boreal regions and 70% of total deposition in the tropical/subtropical regions. Unfortunately, there is a lack of plant and associated Hg litterfall and throughfall deposition data outside of forest ecosystems, such that estimates for global grasslands, savannas, and shrublands remain highly uncertain. Hg stable isotope approaches now suggest that Hg^0^ deposited via plants comprises the majority of Hg deposition to soils in central North America (57–94%) (Demers et al. 2013; Zheng et al. 2016), Alaskan tundra soils (71%) (Obrist et al. 2017), central European peat soils (79%) (Enrico et al. 2016), boreal forest soils in North Sweden (90%) (Jiskra et al. 2015), and high mountain soils in Tibet (Wang et al. 2017). This is likely also true for other areas of the world, for example, the tropics, where some of the highest litterfall Hg deposition is observed (Wang et al. 2016). Recent field studies also show evidence for an active Hg^0^ sink in soils (Sigler and Lee 2006; Moore and Castro 2012; Obrist et al. 2014, 2017) and possibly direct deposition of Hg^0^ to forest litter (Zheng et al. 2016). The finding that litterfall is the dominant terrestrial Hg^0^ source fundamentally reshapes our understanding of Hg deposition to terrestrial landscapes and Hg mobility, demonstrating the need for monitoring strategies to measure net ecosystem Hg^0^ deposition, including direct exchange/deposition measurements. The dominant role of foliar Hg^0^ uptake also results in a specific “terrestrial” Hg isotope signature that can be used to trace terrestrial contributions and transfer to aquatic ecosystems (Tsui et al. 2012; Kwon et al. 2015; Jiskra et al. 2017), aquatic biota (Li et al. 2016), and lake and ocean sediments (Grasby et al. 2015; Lepak et al. 2015; Enrico et al. 2016; Araujo et al. 2017; Gleason et al. 2017). Finally, whole-ecosystem flux measurements using micrometeorological techniques can further confirm the dominant Hg^0^ deposition source to terrestrial ecosystems. The longest data set spans 2 years in the Arctic tundra (Obrist et al. 2017) and shows that Hg^0^ deposition (6.5 ± 0.7 μg/m^2^/year) dominates other deposition processes (71% of total deposition), in agreement with Hg stable isotope analysis. Previous studies over grasslands also reported a net annual dry Hg^0^ deposition [17.5 μg/m^2^/year (Fritsche et al. 2008a) and 3.3 μg/m^2^/year (Castro and Moore 2016)] and shorter-term measurements showed a net Hg^0^ deposition during vegetation periods (Lee et al. 2000; Bash and Miller 2008; Fritsche et al. 2008b; Bash and Miller 2009; Sommar et al. 2016). Other studies, however, have reported inconsistent results, with net terrestrial Hg^0^ emissions even in the presence of vegetation (Lindberg et al. 1998; Cobos et al. 2002; Obrist et al. 2005; Cobbett and Van Heyst 2007; Baya and Van Heyst 2010).

### Atmosphere–aquatic interactions

A new global model suggests that approximately 50% of total global wet Hg^II^ deposition may occur to tropical oceans within 30° northern and southern latitudes due to a high abundance of free tropospheric Hg^II^ reservoirs scavenged by precipitation in these regions (Horowitz et al. 2017). These important global hotspot regions for Hg^II^ deposition need to be confirmed by direct deposition measurements.

Air–sea exchange of Hg^0^ is critical to extending the lifetime of anthropogenic Hg in the atmosphere, terrestrial ecosystems, and the ocean (Strode et al. 2007; Soerensen et al. 2010). The magnitude of net Hg^0^ evasion from the ocean is approximately 2900 Mg/year (range 1900–4200 Mg/year) (Horowitz et al. 2017; Streets et al. 2017), compared to approximately 2000 Mg/year from primary anthropogenic emissions; thus, the ocean is considered the largest source of atmospheric Hg globally. There is, however, substantial uncertainty in the magnitude of air–sea exchange from different ocean regions. Simultaneous measurements of dissolved Hg^0^ in seawater and atmospheric concentrations are used to assess this source, with recent latitudinal gradients in both the Atlantic and Pacific Oceans showing distinct spikes in concentrations around the Intertropical Convergence Zone (ITCZ). These spikes are thought to result from deep convection and intense precipitation in these regions that increase atmospheric Hg^II^ deposition (Soerensen et al. 2014). Using high-frequency measurements of atmospheric and aquatic Hg^0^ concentrations, Soerensen et al. (2013) inferred that terrestrial DOC can inhibit Hg^II^ reduction and ambient Hg^0^ production in seawater. Lamborg et al. (2016) measured concentrations of Hg on settling organic particulate matter in the Atlantic Ocean and suggested that partitioning to particles may be up to 1000 times greater than assumed in some models. This would effectively lower the magnitude of the global evasion flux of Hg^0^ from the ocean because scavenging of Hg from surface waters would reduce the pool of Hg^II^ available for reduction and conversion to Hg^0^.

### Terrestrial–aquatic interactions

In watersheds of the Americas, with historic Hg and precious metal mining, runoff from contaminated sites dominates aquatic Hg loads and is substantial (44 Mg/year) (Kocman et al. 2017). In contrast, in developed regions, including Europe and North America, catchment inputs and atmospheric deposition are generally the primary Hg sources, with background terrestrial environments accounting for approximately 230 Mg/year (170–300 Mg) of riverine loads. Hydrology and DOC mobility are the major controls on delivery of catchment Hg to aquatic ecosystems, with episodic high-flow periods from storms and/or snowmelt often representing the majority of annual catchment inputs (St. Louis et al. 2016).

Recent Hg stable isotope analyses have been able to differentiate among inorganic Hg sources to freshwater. The waters of remote Canadian lakes share the positive Δ^200^Hg signature observed in precipitation, suggesting that atmospheric deposition to these lakes is the major Hg source (42 ± 26%) (Chen et al. 2016). Similarly, analyses of sediment of the Laurentian Great Lakes indicate that atmospheric Hg^II^ deposition is the dominant Hg source to Lakes Huron, Superior, and Michigan, while terrestrial catchment and industrial effluent are the dominant Hg sources to Lakes Erie and Ontario (Jackson et al. 2015; Lepak et al. 2015). Contrasting results have been reported for a boreal forest catchment in Sweden, where Hg stable isotope analyses suggest that Hg in forest runoff originated from the deposition of Hg^0^ through foliar uptake, rather than from precipitation (Jiskra et al. 2017). Analyses of dated lake sediment cores and catchment soils using geochemical tracers can also be used to tease apart changes in atmospheric and terrestrial Hg^II^ inputs over time (Fitzgerald et al. 2005; Perry et al. 2005; Landers et al. 2008; Muir et al. 2009; Kirk et al. 2011; Hermanns and Biester 2013). Recent analyses of Hg stable isotopes in dated lake sediment cores have also been used to examine temporal changes in Hg sources (Sonke et al. 2010; Gehrke et al. 2011; Cooke et al. 2013; Donovan et al. 2013).

For pelagic ocean regions, the dominant source of Hg is atmospheric deposition (Soerensen et al. 2010). Rivers comprise only a small fraction of Hg input to most ocean basins because the majority of Hg is in the particle phase and settles in ocean margins. Zhang et al. (2015b) showed that the fraction of riverine Hg exported to the open ocean is 6–25% for all basins except the Arctic. The large shelf and relatively smaller surface area of the Arctic Ocean make riverine inputs of Hg a more important source. For example, modeling studies have used atmospheric observations to infer a large missing Hg source from rivers and coastal erosion (Fisher et al. 2012; Zhang et al. 2015b). Other work using measured Hg:DOC ratios and a synthesis of available observations suggests a riverine Hg source to the Arctic of approximately 50 Mg/year (Dastoor et al. 2015; Zhang et al. 2015b), which is slightly larger than atmospheric deposition of Hg (approximately 45 Mg/year).

## Anticipated impacts from human and natural perturbations, including emission changes and climate change, on global Hg cycling

As illustrated in Fig. 2, drivers of perturbations on the global Hg cycle include emission, climate, and land use changes.

### Atmosphere

Critical factors affecting future anthropogenic emissions include energy use and Hg emission control strategies [e.g., in response to the Minamata Convention, see Selin et al. 2018 and Giang et al. 2015] and mitigation measures for global climate change. One of the largest anthropogenic sources of Hg, ASGM emissions, will critically affect future Hg emissions, but is also associated with the largest uncertainties in future emission estimates. Streets et al. (2009) projected the range of 2050 global Hg emissions to be 2390–4860 Mg, compared to the 2006 level of 2480 Mg. For combustion sources, large-scale deployment of advanced Hg sorbent technologies, such as activated carbon injection (ACI), may lower the 2050 emission range to 1670–3480 Mg/year. A study by Giang et al. (2015) suggested 90 and 150 Mg/year of avoided power sector emissions for China and India in 2050, respectively, due to Minamata Convention control technology requirements. Declining Hg emissions in China through 2050 could, however, be offset by increasing Indian emissions (Giang et al. 2015), leading to a shift in source areas globally toward the tropics. Rafaj et al. (2013) evaluated the co-benefit of global greenhouse gas mitigation efforts and found that anthropogenic Hg emissions under a global climate mitigation regime would be reduced in 2050 by 45% compared to the case without climate measures. Pacyna et al. (2016) estimated that a best-case scenario of Hg emission decreases by 2050 could result in a decrease in Hg of up to 50% in the northern hemisphere and 35% in the southern hemisphere.

Lei et al. (2014) compared the influence of projected emission changes and climate change on U.S. atmospheric Hg levels in 2050. They found that anthropogenic emissions would contribute 32–53% of projected changes in Hg air concentration, while climate and natural emission changes would account for 47–68%. Of the effects of climate change, influences via secondary emissions and ecosystem structure (see the “Atmosphere–terrestrial interactions” section) will likely exert the largest changes on atmospheric Hg pools. Although less important, atmospheric processes can also be directly impacted by climate change; for example, the oxidation rate constant of the Hg–Br reaction is sensitive to temperature. Zhang et al. (2016a) estimated a 5–7% increase in northern hemisphere atmospheric surface Hg^0^ concentrations by 2050 because of reduced oxidation and increased in-cloud reduction. Changes in precipitation patterns can change spatial distribution, magnitude, and seasonal variation of Hg^II^ deposition. Megaritis et al. (2014) projected increases in Hg^II^ deposition due to climate change to the eastern U.S. of 6% during summer and 4% during winter by 2050, with significant spatial variability. Zhang et al. (2016a) predicted that land use and land cover changes may lead to increases in Hg^0^ dry deposition fluxes under future climate. Hansen et al. (2015) projected climate-induced decreases in Hg deposition to the Arctic, while Zhang et al. (2016a) estimated increases in northern high latitudes. Large-scale meteorological patterns such as El Niño-Southern Oscillation may also influence interannual variability of atmospheric Hg concentrations (Slemr et al. 2016).

### Terrestrial

Major impacts on global Hg cycling are expected due to shifts in global biomes, hydrology, fire patterns, water table depth, soil moisture, and redox conditions. In addition, forestry practices and deforestation can affect watershed Hg processes and MeHg exposures and biomagnification, although responses can be variable between different watersheds (discussed in detail in Hsu-Kim et al. (2018)). Based on the importance of vegetation for atmospheric Hg deposition, predicted shifts in biomes will directly impact terrestrial and aquatic Hg distribution and impacts. Modeling studies have predicted a strong sensitivity of Hg^0^ dry deposition to changes in vegetation (Krabbenhoft and Sunderland 2013; Zhang et al. 2016a). Zhang et al. (2016a) projected that by 2050 the annual mean Hg^0^ dry deposition flux over land will increase by 20% in northern mid-latitudes, driven by a combination of increased atmospheric Hg^0^ exposure and increased vegetation and foliage density induced by CO_2_ fertilization. Regionally decreased Hg^0^ dry deposition may occur in South Asia and Africa, for example, where projected increases of agricultural land area will lead to losses of leafy areas. Strong shifts in soil Hg accumulation have been predicted as well. Hararuk et al. (2013) suggested that, along with changes in soil organic carbon, soil Hg sequestration in the U.S. will increase by 2100, mainly induced by changes in precipitation patterns. They also predicted that increases in CO_2_ concentrations to 700 ppm would stimulate both soil carbon and soil Hg accruals, while increased air temperatures would have small negative effects on both. The combined effects of increased CO_2_, increased temperature, and increased or decreased precipitation will likely lead to pronounced regional differences in soil Hg changes. Anticipated shifts in coniferous versus deciduous forest abundance will also likely impact terrestrial Hg distribution. Richardson and Friedland (2015) suggested that anticipated losses of 2.2 million ha of coniferous forests will lead to 27 Mg less Hg sequestered in organic horizons across this region, either as a function of reduced atmospheric Hg deposition or increased release into watersheds. These anticipated effects of climate change and ecosystem properties on Hg cycling can be seen clearly in archive studies. In a remote lake in Patagonia, for example, pre-anthropogenic changes in sediment loads up to a factor of four were found, comparable to recent anthropogenic forcing (Hermanns et al. 2012), and were attributed to past changes in deposition related to climate-induced shifts in terrestrial organic matter flux and DOM leaching from catchment soils. Similarly, Rydberg et al. (2015) documented how a climate-induced change from deciduous to coniferous forests around 5700 years BP led to reduced soil erosion and increased transport of DOC-bound Hg from a catchment in the area of Germany, resulting in increased Hg concentrations and accumulation rates.

Other ecosystem disturbances are expected to affect Hg cycling, but in unknown directions. For example, changes in wildfire frequency and abundance will impact atmospheric Hg emissions and watershed fluxes in a nonlinear fashion given the complex relationships between Hg emissions and fire intensities, pre-fire Hg accumulation, and post-fire Hg mobilization. Wildfire Hg emissions now likely include substantial emissions of anthropogenic legacy Hg accumulated in litter and soils, and climate and land use change impacts on fire frequency and activity need to be considered as part of human impacts (Westerling et al. 2006). A recent modeling study (Kumar et al. 2017) estimated a 14% increase in wildfire-related Hg emission due to climate change. Corresponding land use changes may amplify or alleviate this effect, e.g., due to reductions in forest fires by agricultural land expansion in Africa or increased forest abundance in North America and Asia. In addition, land management practices such as burning of agricultural fields, grassland, and brush fires contribute to Hg emissions, although little information is available on their global contribution. Land use changes such as shifts in land management and forestry practices also have the potential to mobilize terrestrial Hg pools, via increased erosion, changes in hydrological pathways, and changes in yields (Kocman et al. 2017; Kronberg et al. 2016). Kocman et al. (2017) summarized studies that show pronounced Hg leaching when soils are converted to agricultural land and predicted that global deforestation of 13 million ha/yr in the 2000s may have yielded up to 260 Mg Hg per year entering local freshwaters.

### Aquatic systems

Both oceans and freshwater ecosystems will be affected by changing Hg emissions and climate-induced alterations. Modeling studies show that response times for lakes can range from a few years to many decades (Harris et al. 2007; Knightes et al. 2009). Recovery of freshwater ecosystems to decreased atmospheric Hg emissions is expected when reduced atmospheric Hg loadings translates into a substantial decrease in DOC-bound MeHg and inorganic Hg catchment runoff (Graham et al. 2013; Chiasson-Gould et al. 2014; French et al. 2014; Jonsson et al. 2014; Lepak et al. 2015; Ndu et al. 2015). A recent long-term mass balance study in New York state suggested that Hg inputs to lakes from forested catchments could be very responsive to decreased Hg emissions (Gerson and Driscoll 2016). They reported that a 25% reduction in atmospheric Hg^0^ concentration resulted in a 40% reduction in litterfall Hg fluxes, which in turn decreased inorganic Hg and MeHg stream inputs (45 and 29%, respectively) and lake concentrations over a 10-year period (Gerson and Driscoll 2016).

A common disturbance of freshwater Hg cycling results from widespread reservoir creation, which generally amplifies MeHg production; reservoir effects are discussed in detail in Eagles-Smith et al. (2018) and Hsu-Kim et al. (2018). Most studies of climate change impacts on freshwater Hg cycling have been conducted in sensitive ecosystems experiencing accelerated changes (e.g., high-altitude and high-latitude lakes, ponds, and peatlands). In the western Canadian Arctic, where approximately 15% of the water bodies have shoreline retrogressive thaw slump lakes, lake sediment cores demonstrated increased sedimentation of inorganics and decreased sediment concentrations of DOC, THg, and MeHg (Deison et al. 2012). In contrast, a survey of MeHg accumulation in invertebrates in tundra lakes suggested that lakes with low DOC are experiencing increased DOC input because of humification and these lakes may see a two- to threefold increase in Hg bioaccumulation as the DOC concentration approaches a threshold of approximately 8 mg/L (MacMillan et al. 2015). Jonsson et al. (2017) predicted a three- to sixfold increase in MeHg concentration in estuarine zooplankton in association with large inputs of terrestrial DOC intended to mimic potential future changes in runoff. Peat mesocosm experiments suggest that changes in hydrological regimes and shifts in vascular plant communities may have a significant impact on Hg cycling in peatlands (Haynes 2017). For example, lower, more variable water tables and the removal of Ericaceae shrubs significantly enhanced inorganic Hg and MeHg mobility in peat pore waters and MeHg export from snowmelt, likely from enhanced peat decomposition and internal regeneration of electron acceptors related to water table changes (Haynes et al. 2017).

The effect on Hg of large-scale changes in the marine environment expected from climate change was explored by Krabbenhoft and Sunderland (2013). Increased seawater temperatures may enhance organic matter remineralization and the propensity for MeHg production in some regions of the ocean. Rapid changes in sea-ice cover and seasonality in the polar oceans are likely to exert a major impact on air–sea exchange of Hg in these regions, and further work may be needed to understand how temperature and sea-ice dynamics alter Hg dynamics in the Arctic (Angot et al. 2016). Changes in microbial community structure and ocean productivity will propagate through marine food webs in potentially unexpected ways, altering bioaccumulation. Fisher et al. (2013) used 30 years of satellite data to quantify the impacts of interannual variability in meteorology on inorganic Hg dynamics in the Arctic. They concluded that the dominant climate mode of the future in the Arctic may result in a lower reservoir of Hg in the Arctic Ocean because of enhanced air–sea exchange. This work did not consider the effects of a changing (melting) terrestrial landscape, which may dramatically increase Hg input to the Arctic from the terrestrial environment. Soerensen et al. (2016) suggested that rivers are likely to become more dominant sources of Hg and MeHg in the future Arctic Ocean. Stern et al. (2012) reviewed a variety of changes that are occurring and expected to occur in the Arctic Ocean in the future, and suggested that future effects are likely to be large; they also highlighted current limitations in understanding multiple, simultaneously occurring impacts on Hg cycling and bioaccumulation. Finally, recent modeling suggests that the response times of marine fish tissue burdens to changes in Hg input will depend on the locations of MeHg production (Li et al. 2016). Benthic sediment will respond much more slowly than the upper Ocean and estuarine surface waters (Sunderland et al. 2010; Schartup et al. 2015; Soerensen et al. 2016). Amos et al. (2013) predicted that legacy Hg will be present in global ecosystems for periods ranging from decades to millennia, suggesting a large time lag in response to changes in anthropogenic emissions and climate.

## Conclusions and remaining uncertainties

We reviewed how our understanding of Hg global cycling has advanced in the last decade, focusing on environmental reservoirs and processes within and between these reservoirs. With emerging large global datasets, in combination with improved models and analytical techniques, new constraints are possible on the magnitude of reservoirs and fluxes. In the atmosphere, while Hg^0^ concentrations and wet deposition are decreasing in some parts of Europe and North America, other regions such as Asia show increasing trends. Updated knowledge about the presence and chemistry of Hg^II^, especially in the free troposphere, suggests a source of global Hg to the tropics. In terrestrial ecosystems, updated information gained from new measurements and models has changed our understanding of Hg exchange between terrestrial systems and the ocean. Previous estimates of a large terrestrial source of Hg to the atmosphere have been revised downward, and new data suggest that Hg^0^ uptake is dominant in natural systems. In aquatic systems, recent research has shown the potential importance of direct Hg releases to water in both freshwater and marine systems. In oceans, new constraints are available on the total amount of Hg in ocean systems and its distribution and trends, with declines in the Atlantic and possible increases in the Pacific.

Global changes, including climate, land use, and Hg emissions changes, are likely to profoundly alter Hg cycling. New analyses have indicated the direction of these possible changes. Global policy such as that emerging from the Minamata Convention may avoid significant increases in Hg concentrations associated with future economic growth; however, legacy emissions will continue to affect the global cycle for decades to centuries. Projections of future conditions are becoming more quantitative, supported by increasingly large and globally linked datasets, new tools, and better models. Global changes are also expected to affect concentrations of MeHg in potentially nonlinear ways, including changes in methylation and food web bioaccumulation [see Eagles-Smith et al. (2018) and Hsu-Kim et al. (2018)].

There remain uncertainties that limit our ability to predict future global cycling and therefore human exposure, especially in the context of multiple and simultaneous changes. While data availability has increased, several regions of the globe, especially the tropics and the southern hemisphere, remain understudied. In the atmosphere, uncertainties in atmospheric chemistry and in measurements of Hg^II^ challenge our quantitative understanding of oxidation pathways. Advances and wider applications in isotope techniques are needed to better fingerprint specific sources and mechanisms. In terrestrial ecosystems, time-extended measurements of Hg^0^ uptake, now known to be a significant input process, are largely lacking. Finally, the global Hg cycle will also depend on the choices made by governments both now and in the future, including policy efforts to mitigate emissions of Hg and control climate change.

## References

[CR1] Agnan Y, Le Dantec T, Moore CW, Edwards GC, Obrist D (2016). New constraints on terrestrial surface-atmosphere fluxes of gaseous elemental mercury using a global database. Environmental Science and Technology.

[CR2] AMAP/UNEP. 2008. Technical Background Report to the Global Atmospheric Mercury Assessment. Arctic Monitoring and Assessment Programme and United Nations Environment Programme, Geneva, Switzerland

[CR3] AMAP/UNEP. 2013. Technical Background Report for the Global Mercury Assessment 2013. Arctic Monitoring and Assessment Programme and United Nations Environment Programme, Geneva, Switzerland

[CR5] Amos HM, Jacob DJ, Streets DG, Sunderland EM (2013). Legacy impacts of all-time anthropogenic emissions on the global mercury cycle. Global Biogeochemical Cycles.

[CR4] Amos HM, Jacob DJ, Kocman D, Horowitz HM, Zhang Y, Dutkiewicz S, Horvat M, Corbitt ES (2014). Global biogeochemical implications of mercury discharges from rivers and sediment burial. Environmental Science and Technology.

[CR6] Amos HM, Sonke JE, Obrist D, Robins N, Hagan N, Horowitz HM, Mason RP, Witt M (2015). Observational and modeling constraints on global anthropogenic enrichment of mercury. Environmental Science and Technology.

[CR7] Angot H, Dastoor A, De Simone F, Gårdfeldt K, Gencarelli CN, Hedgecock IM, Langer S, Magand O (2016). Chemical cycling and deposition of atmospheric mercury in polar regions: Review of recent measurements and comparison with models. Atmospheric Chemistry and Physics.

[CR8] Araujo BF, Hintelmann H, Dimock B, Almeida MG, Rezende CE (2017). Concentrations and isotope ratios of mercury in sediments from shelf and continental slope at Campos Basin near Rio de Janeiro, Brazil. Chemosphere.

[CR9] Bae HS, Dierberg FE, Ogram A (2014). Syntrophs dominate sequences associated with the mercury methylation-related gene hgcA in the water conservation areas of the Florida Everglades. Applied and Environment Microbiology.

[CR11] Bash JO, Miller DR (2008). A relaxed eddy accumulation system for measuring surface fluxes of total gaseous mercury. Journal of Atmospheric and Oceanic Technology.

[CR12] Bash JO, Miller DR (2009). Growing season total gaseous mercury (TGM) flux measurements over an *Acer rubrum* L. stand. Atmospheric Environment.

[CR10] Bash JO, Bresnahan P, Miller DR (2007). Dynamic surface interface exchanges of mercury: A review and compartmentalized modeling framework. Journal of Applied Meteorology and Climatology.

[CR13] Baya AP, Van Heyst B (2010). Assessing the trends and effects of environmental parameters on the behaviour of mercury in the lower atmosphere over cropped land over four seasons. Atmospheric Chemistry and Physics.

[CR14] Benoit JM, Gilmour CC, Mason RP, Heyes A (1999). Sulfide controls on mercury speciation and bioavailability to methylating bacteria in sediment pore waters. Environmental Science and Technology.

[CR15] Bergquist BA, Blum JD (2007). Mass-dependent and -independent fractionation of Hg isotopes by photoreduction in aquatic systems. Science.

[CR16] Bieser, J., F. Slemr, J. Ambrose, C. Brenninkmeijer, S. Brooks, A. Dastoor, F. DeSimone, R. Ebinghaus, et al. 2017. Multi-model study of mercury dispersion in the atmosphere: Vertical and interhemispheric distribution of mercury species. *Atmospheric Chemistry and Physics* 17: 6925–6955.

[CR17] Biester H, Bindler R, Martinez-Cortizas A, Engstrom DR (2007). Modeling the past atmospheric deposition of mercury using natural archives. Environmental Science and Technology.

[CR18] Bigeleisen J (1996). Nuclear size and shape effects in chemical reactions. Isotope chemistry of the heavy elements. Journal of the American Chemical Society.

[CR20] Biswas A, Blum JD, Klaue B, Keeler GJ (2007). Release of mercury from Rocky Mountain forest fires. Global Biogeochemical Cycles.

[CR19] Biswas A, Blum JD, Keeler GJ (2008). Mercury storage in surface soils in a central Washington forest and estimated release during the 2001 Rex Creek Fire. Science of the Total Environment.

[CR21] Blackwell BD, Driscoll CT (2015). Deposition of mercury in forests along a montane elevation gradient. Environmental Science and Technology.

[CR22] Blais JM, Rosen M, Smol JP (2015). Environmental contaminants: Using natural archives to track sources and long-term trends of pollution.

[CR23] Blum JD, Bergquist BA (2007). Reporting of variations in the natural isotopic composition of mercury. Analytical and Bioanalytical Chemistry.

[CR24] Blum JD, Johnson MW (2017). Recent developments in mercury stable isotope analysis. Reviews in Mineralogy and Geochemistry.

[CR25] Blum JD, Popp BN, Drazen JC, Anela Choy C, Johnson MW (2013). Methylmercury production below the mixed layer in the North Pacific Ocean. Nature Geoscience.

[CR26] Blum JD, Sherman LS, Johnson MW (2014). Mercury isotopes in earth and environmental sciences. Annual Review of Earth and Planetary Sciences.

[CR27] Boening DW (2000). Ecological effects, transport, and fate of mercury: A general review. Chemosphere.

[CR29] Bowman KL, Hammerschmidt CR, Lamborg CH, Swarr G (2014). Mercury in the North Atlantic Ocean: The US GEOTRACES zonal and meridional sections. Deep Sea Research Part II: Topical Studies in Oceanography.

[CR30] Bowman KL, Hammerschmidt CR, Lamborg CH, Swarr GJ, Agather AM (2016). Distribution of mercury species across a zonal section of the eastern tropical South Pacific Ocean (U.S. GEOTRACES GP16). Marine Chemistry.

[CR31] Bravo AG, Bouchet S, Guedron S, Amouroux D, Dominik J, Zopfi J (2015). High methylmercury production under ferruginous conditions in sediments impacted by sewage treatment plant discharges. Water Research.

[CR32] Buchachenko AL (2001). Magnetic isotope effect: Nuclear spin control of chemical reactions. The Journal of Physical Chemistry A.

[CR33] Buchachenko AL (2009). Mercury isotope effects in the environmental chemistry and biochemistry of mercury-containing compounds. Russian Chemical reviews.

[CR34] Burger Chakraborty L, Qureshi A, Vadenbo C, Hellweg S (2013). Anthropogenic mercury flows in India and impacts of emission controls. Environmental Science and Technology.

[CR35] Burke MP, Hogue TS, Ferreira M, Mendez CB, Navarro B, Lopez S, Jay JA (2010). The effect of wildfire on soil mercury concentrations in Southern California watersheds. Water, Air, and Soil Pollution.

[CR36] Butler TJ, Cohen MD, Vermeylen FM, Likens GE, Schmeltz D, Artz RS (2008). Regional precipitation mercury trends in the eastern USA, 1998–2005: Declines in the Northeast and Midwest, no trend in the Southeast. Atmospheric Environment.

[CR37] Castro M, Moore C (2016). Importance of gaseous elemental mercury fluxes in western Maryland. Atmosphere.

[CR38] Chen J, Hintelmann H, Feng X, Dimock B (2012). Unusual fractionation of both odd and even mercury isotopes in precipitation from Peterborough, ON, Canada. Geochimica et Cosmochimica Acta.

[CR39] Chen J, Hintelmann H, Zheng W, Feng X, Cai H, Wang Z, Yuan S, Wang Z (2016). Isotopic evidence for distinct sources of mercury in lake waters and sediments. Chemical Geology.

[CR40] Chiasson-Gould SA, Blais JM, Poulain AJ (2014). Dissolved organic matter kinetically controls mercury bioavailability to bacteria. Environmental Science and Technology.

[CR41] Choi HD, Holsen TM (2009). Gaseous mercury fluxes from the forest floor of the Adirondacks. Environmental Pollution.

[CR42] Christensen GA, Wymore AM, King AJ, Podar M, Hurt RA, Santillan EU, Soren A, Brandt CC (2016). Development and validation of broad-range qualitative and clade-specific quantitative molecular probes for assessing mercury methylation in the environment. Applied and Environment Microbiology.

[CR43] Cobbett FD, Van Heyst BJ (2007). Measurements of GEM fluxes and atmospheric mercury concentrations (GEM, RGM and Hg-P) from an agricultural field amended with biosolids in Southern Ont., Canada (October 2004–November 2004). Atmospheric Environment.

[CR44] Cobos D, Baker J, Nater E (2002). Conditional sampling for measuring mercury vapor fluxes. Atmospheric Environment.

[CR46] Cole AS, Steffen A, Pfaffhuber KA, Berg T, Pilote M, Poissant L, Tordon R, Hung H (2013). Ten-year trends of atmospheric mercury in the high Arctic compared to Canadian sub-Arctic and mid-latitude sites. Atmospheric Chemistry and Physics.

[CR45] Cole A, Steffen A, Eckley C, Narayan J, Pilote M, Tordon R, Graydon J, St Louis VL (2014). A survey of mercury in air and precipitation across Canada: Patterns and trends. Atmosphere.

[CR47] Cooke CA, Hintelmann H, Ague JJ, Burger R, Biester H, Sachs JP, Engstrom DR (2013). Use and legacy of mercury in the Andes. Environmental Science and Technology.

[CR50] Cossa D, Martin J-M, Takayanagi K, Sanjuan J (1997). The distribution and cycling of mercury species in the western Mediterranean. Deep Sea Research Part II: Topical Studies in Oceanography.

[CR48] Cossa D, Averty B, Pirrone N (2009). The origin of methylmercury in open Mediterranean waters. Limnology and Oceanography.

[CR49] Cossa D, Heimbürger L-E, Lannuzel D, Rintoul SR, Butler ECV, Bowie AR, Averty B, Watson RJ (2011). Mercury in the Southern Ocean. Geochimica et Cosmochimica Acta.

[CR52] Dastoor AP, Larocque Y (2004). Global circulation of atmospheric mercury: A modelling study. Atmospheric Environment.

[CR51] Dastoor A, Ryzhkov A, Durnford D, Lehnherr I, Steffen A, Morrison H (2015). Atmospheric mercury in the Canadian Arctic. Part II: Insight from modeling. Science of the Total Environment.

[CR53] De Simone F, Cinnirella S, Gencarelli CN, Yang X, Hedgecock IM, Pirrone N (2015). Model study of global mercury deposition from biomass burning. Environmental Science and Technology.

[CR54] Deison R, Smol JP, Kokelj SV, Pisaric MF, Kimpe LE, Poulain AJ, Sanei H, Thienpont JR (2012). Spatial and temporal assessment of mercury and organic matter in thermokarst affected lakes of the Mackenzie Delta uplands, NT, Canada. Environmental Science and Technology.

[CR55] Demers JD, Blum JD, Zak DR (2013). Mercury isotopes in a forested ecosystem: Implications for air-surface exchange dynamics and the global mercury cycle. Global Biogeochemical Cycles.

[CR56] Demers JD, Sherman LS, Blum JD, Marsik FJ, Dvonch JT (2015). Coupling atmospheric mercury isotope ratios and meteorology to identify sources of mercury impacting a coastal urban-industrial region near Pensacola, Florida, USA. Global Biogeochemical Cycles.

[CR57] Denzler B, Bogdal C, Henne S, Obrist D, Steinbacher M, Hungerbuhler K (2017). Inversion approach to validate mercury emissions based on background air monitoring at the high altitude research station Jungfraujoch (3580 m). Environmental Science and Technology.

[CR58] Dibble TS, Zelie MJ, Mao H (2012). Thermodynamics of reactions of ClHg and BrHg radicals with atmospherically abundant free radicals. Atmospheric Chemistry and Physics.

[CR59] Donovan PM, Blum JD, Yee D, Gehrke GE, Singer MB (2013). An isotopic record of mercury in San Francisco Bay sediment. Chemical Geology.

[CR60] Drevnick PE, Cooke CA, Barraza D, Blais JM, Coale KH, Cumming BF, Curtis CJ, Das B (2016). Spatiotemporal patterns of mercury accumulation in lake sediments of western North America. Science of the Total Environment.

[CR61] Driscoll CT, Han YJ, Chen CY, Evers DC, Lambert KF, Holsen TM, Kamman NC, Munson RK (2007). Mercury contamination in forest and freshwater ecosystems in the Northeastern United States. BioScience.

[CR62] Du H, Ma M, Sun T, Dai X, Yang C, Luo F, Wang D, Igarashi Y (2017). Mercury-methylating genes dsrB and hgcA in soils/sediments of the Three Gorges Reservoir. Environmental Science and Pollution Research International.

[CR63] Durnford D, Dastoor A, Ryzhkov A, Poissant L, Pilote M, Figueras-Nieto D (2012). How relevant is the deposition of mercury onto snowpacks? Part 2: A modeling study. Atmospheric Chemistry and Physics.

[CR64] Eagles-Smith, C.A., E.K. Silbergeld, N. Basu, P. Bustamante, F. Diaz-Barriga, W.A. Hopkins, K.A. Kidd, and J.F. Nyland. 2018. Modulators of mercury risk to wildlife and humans in the context of rapid global change. *Ambio*. 10.1007/s13280-017-1011-x. 10.1007/s13280-017-1011-x PMC5794686 29388128

[CR65] Ebinghaus R, Jennings SG, Kock HH, Derwent RG, Manning AJ, Spain TG (2011). Decreasing trends in total gaseous mercury observations in baseline air at Mace Head, Ireland from 1996 to 2009. Atmospheric Environment.

[CR66] Eckley CS, Tate MT, Lin CJ, Gustin M, Dent S, Eagles-Smith C, Lutz MA, Wickland KP (2016). Surface-air mercury fluxes across Western North America: A synthesis of spatial trends and controlling variables. Science of the Total Environment.

[CR67] Engle MA, Sexauer Gustin M, Johnson DW, Murphy JF, Miller WW, Walker RF, Wright J, Markee M (2006). Mercury distribution in two Sierran forest and one desert sagebrush steppe ecosystems and the effects of fire. Science of the Total Environment.

[CR69] Engstrom DR, Swain EB (1997). Recent declines in atmospheric mercury deposition in the upper midwest. Environmental Science and Technology.

[CR68] Engstrom DR, Fitzgerald WF, Cooke CA, Lamborg CH, Drevnick PE, Swain EB, Balogh SJ, Balcom PH (2014). Atmospheric Hg emissions from preindustrial gold and silver extraction in the Americas: A reevaluation from lake-sediment archives. Environmental Science and Technology.

[CR71] Enrico M, Roux GL, Marusczak N, Heimburger LE, Claustres A, Fu X, Sun R, Sonke JE (2016). Atmospheric mercury transfer to peat bogs dominated by gaseous elemental mercury dry deposition. Environmental Science and Technology.

[CR70] Enrico M, Le Roux G, Heimburger LE, Van Beek P, Souhaut M, Chmeleff J, Sonke JE (2017). Holocene atmospheric mercury levels reconstructed from peat bog mercury stable isotopes. Environmental Science and Technology.

[CR72] Ericksen JA, Gustin MS, Xin M, Weisberg PJ, Fernandez GC (2006). Air-soil exchange of mercury from background soils in the United States. Science of the Total Environment.

[CR73] Faïn X, Ferrari CP, Dommergue A, Albert MR, Battle M, Severinghaus J, Arnaud L, Barnola JM (2009). Polar firn air reveals large-scale impact of anthropogenic mercury emissions during the 1970s. Proceedings of the National academy of Sciences of the United States of America.

[CR74] Farmer JG, Anderson P, Cloy JM, Graham MC, MacKenzie AB, Cook GT (2009). Historical accumulation rates of mercury in four Scottish ombrotrophic peat bogs over the past 2000 years. Science of the Total Environment.

[CR76] Fisher JA, Jacob DJ, Soerensen AL, Amos HM, Steffen A, Sunderland EM (2012). Riverine source of Arctic Ocean mercury inferred from atmospheric observations. Nature Geoscience.

[CR75] Fisher JA, Jacob DJ, Soerensen AL, Amos HM, Corbitt ES, Streets DG, Wang Q, Yantosca RM (2013). Factors driving mercury variability in the Arctic atmosphere and ocean over the past 30 years. Global Biogeochemical Cycles.

[CR77] Fitzgerald WF, Engstrom DR, Lamborg CH, Tseng CM, Balcom PH, Hammerschmidt CR (2005). Modern and historic atmospheric mercury fluxes in northern Alaska: Global sources and Arctic depletion. Environmental Science and Technology.

[CR78] Fitzgerald WF, Lamborg CH, Hammerschmidt CR (2007). Marine biogeochemical cycling of mercury. Chemical Reviews.

[CR79] Fleck JA, Marvin-DiPasquale M, Eagles-Smith CA, Ackerman JT, Lutz MA, Tate M, Alpers CN, Hall BD (2016). Mercury and methylmercury in aquatic sediment across western North America. Science of the Total Environment.

[CR80] French TD, Houben AJ, Desforges JP, Kimpe LE, Kokelj SV, Poulain AJ, Smol JP, Wang X (2014). Dissolved organic carbon thresholds affect mercury bioaccumulation in Arctic lakes. Environmental Science and Technology.

[CR81] Friedli HR, Radke LF, Lu JY (2001). Mercury in smoke from biomass fires. Geophysical Research Letters.

[CR82] Friedli HR, Radke LF, Lu JY, Banic CM, Leaitch WR, MacPherson JI (2003). Mercury emissions from burning of biomass from temperate North American forests: Laboratory and airborne measurements. Atmospheric Environment.

[CR83] Fritsche J, Obrist D, Zeeman M, Conen F, Eugster W, Alewell C (2008). Elemental mercury fluxes over a sub-alpine grassland determined with two micrometeorological methods. Atmospheric Environment.

[CR84] Fritsche J, Wohlfahrt G, Ammann C, Zeeman M, Hammerle A, Obrist D, Alewell C (2008). Summertime elemental mercury exchange of temperate grasslands on an ecosystem-scale. Atmospheric Chemistry and Physics.

[CR86] Fu X, Yang X, Lang X, Zhou J, Zhang H, Yu B, Yan H, Lin C-J (2016). Atmospheric wet and litterfall mercury deposition at urban and rural sites in China. Atmospheric Chemistry and Physics.

[CR87] Fu X, Zhu W, Zhang H, Sommar J, Yu B, Yang X, Wang X, Lin C-J (2016). Depletion of atmospheric gaseous elemental mercury by plant uptake at Mt. Changbai, Northeast China. Atmospheric Chemistry and Physics.

[CR88] Gehrke GE, Blum JD, Marvin-DiPasquale M (2011). Sources of mercury to San Francisco Bay surface sediment as revealed by mercury stable isotopes. Geochimica et Cosmochimica Acta.

[CR89] Gerson JR, Driscoll CT (2016). Is mercury in a remote forested watershed of the Adirondack mountains responding to recent decreases in emissions?. Environmental Science and Technology.

[CR90] Giang A, Stokes LC, Streets DG, Corbitt ES, Selin NE (2015). Impacts of the Minamata convention on mercury emissions and global deposition from coal-fired power generation in Asia. Environmental Science and Technology.

[CR91] Gilmour CC, Podar M, Bullock AL, Graham AM, Brown SD, Somenahally AC, Johs A, Hurt RA (2013). Mercury methylation by novel microorganisms from new environments. Environmental Science and Technology.

[CR92] Gionfriddo CM, Tate MT, Wick RR, Schultz MB, Zemla A, Thelen MP, Schofield R, Krabbenhoft DP (2016). Microbial mercury methylation in Antarctic sea ice. Nature Microbiology.

[CR93] Gleason JD, Blum JD, Moore TC, Polyak L, Jakobsson M, Meyers PA, Biswas A (2017). Sources and cycling of mercury in the paleo Arctic Ocean from Hg stable isotope variations in Eocene and Quaternary sediments. Geochimica et Cosmochimica Acta.

[CR95] Goodsite ME, Plane JM, Skov H (2004). A theoretical study of the oxidation of Hg0 to HgBr2 in the troposphere. Environmental Science and Technology.

[CR94] Goodsite ME, Outridge PM, Christensen JH, Dastoor A, Muir D, Travnikov O, Wilson S (2013). How well do environmental archives of atmospheric mercury deposition in the Arctic reproduce rates and trends depicted by atmospheric models and measurements?. Science of the Total Environment.

[CR96] Graham AM, Aiken GR, Gilmour CC (2013). Effect of dissolved organic matter source and character on microbial Hg methylation in Hg-S-DOM solutions. Environmental Science and Technology.

[CR97] Grasby SE, Beauchamp B, Bond DPG, Wignall PB, Sanei H (2015). Mercury anomalies associated with three extinction events (Capitanian Crisis, Latest Permian Extinction and the Smithian/Spathian Extinction) in NW Pangea. Geological Magazine.

[CR99] Gratz LE, Keeler GJ, Blum JD, Sherman LS (2010). Isotopic composition and fractionation of mercury in Great Lakes precipitation and ambient air. Environmental Science and Technology.

[CR98] Gratz LE, Ambrose JL, Jaffe DA, Shah V, Jaeglé L, Stutz J, Festa J, Spolaor M (2015). Oxidation of mercury by bromine in the subtropical Pacific free troposphere. Geophysical Research Letters.

[CR100] Graydon JA, St Louis VL, Lindberg SE, Sandilands KA, Rudd JWM, Kelly CA, Harris R, Tate MT (2012). The role of terrestrial vegetation in atmospheric Hg deposition: Pools and fluxes of spike and ambient Hg from the METAALICUS experiment. Global Biogeochemical Cycles.

[CR101] Gribble MO, Karimi R, Feingold BJ, Nyland JF, O’Hara TM, Gladyshev MI, Chen CY (2016). Mercury, selenium and fish oils in marine food webs and implications for human health. Journal of Marine Biological Association UK.

[CR102] Grigal DF (2003). Mercury sequestration in forests and peatlands: A review. Journal of Environmental Quality.

[CR103] Grigal DF, Kolka RK, Fleck JA, Nater EA (2000). Mercury budget of an upland-peatland watershed. Biogeochemistry.

[CR104] Guedron S, Grimaldi C, Chauvel C, Spadini L, Grimaldi M (2006). Weathering versus atmospheric contributions to mercury concentrations in French Guiana soils. Applied Geochemistry.

[CR106] Gustin MS, Lindberg SE, Austin K, Coolbaugh M, Vette A, Zhang H (2000). Assessing the contribution of natural sources to regional atmospheric mercury budgets. Science of the Total Environment.

[CR107] Gustin MS, Lyman SN, Kilner P, Prestbo E (2011). Development of a passive sampler for gaseous mercury. Atmospheric Environment.

[CR105] Gustin MS, Amos HM, Huang J, Miller MB, Heidecorn K (2015). Measuring and modeling mercury in the atmosphere: A critical review. Atmospheric Chemistry and Physics.

[CR108] Hammerschmidt CR, Bowman KL (2012). Vertical methylmercury distribution in the subtropical North Pacific Ocean. Marine Chemistry.

[CR109] Hansen KM, Christensen JH, Brandt J (2015). the influence of climate change on atmospheric deposition of mercury in the arctic: A model sensitivity study. International Journal of Environmental Research and Public Health.

[CR110] Hararuk O, Obrist D, Luo Y (2013). Modeling the sensitivity of soil mercury storage to climate-induced changes in soil carbon pools. Biogeosciences.

[CR111] Harris RC, Rudd JW, Amyot M, Babiarz CL, Beaty KG, Blanchfield PJ, Bodaly RA, Branfireun BA (2007). Whole-ecosystem study shows rapid fish-mercury response to changes in mercury deposition. Proceedings of the National Academy of Sciences.

[CR112] Haynes, K. M. 2017. Climate change impacts on mercury cycling in peatlands. Ph.D. thesis. University of Toronto, Ontario, Canada.

[CR113] Haynes KM, Kane ES, Potvin L, Lilleskov EA, Kolka RK, Mitchell CPJ (2017). Mobility and transport of mercury and methylmercury in peat as a function of changes in water table regime and plant functional groups. Global Biogeochemical Cycles.

[CR114] Heimbürger L-E, Cossa D, Marty J-C, Migon C, Averty B, Dufour A, Ras J (2010). Methyl mercury distributions in relation to the presence of nano- and picophytoplankton in an oceanic water column (Ligurian Sea, North-western Mediterranean). Geochimica et Cosmochimica Acta.

[CR115] Heimburger LE, Sonke JE, Cossa D, Point D, Lagane C, Laffont L, Galfond BT, Nicolaus M (2015). Shallow methylmercury production in the marginal sea ice zone of the central Arctic Ocean. Science Report.

[CR117] Hermanns YM, Biester H (2013). Anthropogenic mercury signals in lake sediments from southernmost Patagonia, Chile. Science of the Total Environment.

[CR116] Hermanns Y-M, Cortizas AM, Arz H, Stein R, Biester H (2012). Untangling the influence of in-lake productivity and terrestrial organic matter flux on 4,250 years of mercury accumulation in Lake Hambre, Southern Chile. Journal of Paleolimnology.

[CR118] Hintelmann H, Harris R, Heyes A, Hurley JP, Kelly CA, Krabbenhoft DP, Lindberg S, Rudd JW (2002). Reactivity and mobility of new and old mercury deposition in a boreal forest ecosystem during the first year of the METAALICUS study. Mercury experiment to assess atmospheric loading in Canada and the US. Environmental Science and Technology.

[CR119] Holmes CD, Jacob DJ, Corbitt ES, Mao J, Yang X, Talbot R, Slemr F (2010). Global atmospheric model for mercury including oxidation by bromine atoms. Atmospheric Chemistry and Physics.

[CR120] Horowitz HM, Jacob DJ, Amos HM, Streets DG, Sunderland EM (2014). Historical mercury releases from commercial products: Global environmental implications. Environmental Science and Technology.

[CR121] Horowitz HM, Jacob DJ, Zhang Y, Dibble TS, Slemr F, Amos HM, Schmidt JA, Corbitt ES (2017). A new mechanism for atmospheric mercury redox chemistry: Implications for the global mercury budget. Atmospheric Chemistry and Physics.

[CR123] Hsu-Kim H, Kucharzyk KH, Zhang T, Deshusses MA (2013). Mechanisms regulating mercury bioavailability for methylating microorganisms in the aquatic environment: A critical review. Environmental Science and Technology.

[CR122] Hsu-Kim, H., C.S. Eckley, D. Achá, X. Feng, C.C. Gilmour, S. Jonsson, and C.P.J. Mitchell. 2018. Challenges and opportunities for managing aquatic mercury pollution in altered landscapes. *Ambio*. 10.1007/s13280-017-1006-7. 10.1007/s13280-017-1006-7 PMC5794684 29388127

[CR124] Huang J, Gustin MS (2015). Use of passive sampling methods and models to understand sources of mercury deposition to high elevation sites in the Western United States. Environmental Science and Technology.

[CR125] Hynes A, Donohoue D, Goodsite M, Hedgecock I, Mason R, Pirrone N (2009). Our current understanding of major chemical and physical processes affecting mercury dynamics in the atmosphere and at the air-water/terrestrial interfaces. Mercury fate and transport in the global atmosphere.

[CR126] Jackson AK, Evers DC, Adams EM, Cristol DA, Eagles-Smith C, Edmonds ST, Gray CE, Hoskins B (2015). Songbirds as sentinels of mercury in terrestrial habitats of eastern North America. Ecotoxicology.

[CR800] Jaffe, D.A., S. Lyman, H.M. Amos, M.S. Gustin, J. Huang, N.E. Selin, L. Levin, A. ter Schure, et al. 2014. Progress on understanding atmospheric mercury hampered by uncertain measurements. *Environmental Science and Technology* 48: 7204–7206. 10.1021/es5026432 24940613

[CR127] Jiao Y, Dibble TS (2017). First kinetic study of the atmospherically important reactions BrHg + NO_2_ and BrHg + HOO. Physical Chemistry Chemical Physics: PCCP.

[CR128] Jiskra M, Wiederhold JG, Skyllberg U, Kronberg RM, Hajdas I, Kretzschmar R (2015). Mercury deposition and re-emission pathways in boreal forest soils investigated with Hg isotope signatures. Environmental Science and Technology.

[CR129] Jiskra M, Wiederhold JG, Skyllberg U, Kronberg RM, Kretzschmar R (2017). Source tracing of natural organic matter bound mercury in boreal forest runoff with mercury stable isotopes. Environmental Science: Process and Impacts.

[CR131] Jonsson S, Skyllberg U, Nilsson MB, Lundberg E, Andersson A, Bjorn E (2014). Differentiated availability of geochemical mercury pools controls methylmercury levels in estuarine sediment and biota. Nature Communications.

[CR130] Jonsson S, Andersson A, Nilsson MB, Skyllberg U, Lundberg E, Schaefer JK, Akerblom S, Bjorn E (2017). Terrestrial discharges mediate trophic shifts and enhance methylmercury accumulation in estuarine biota. Science Advances.

[CR132] Jung G, Hedgecock IM, Pirrone N (2009). ECHMERIT V1.0: A new global fully coupled mercury-chemistry and transport model. Geoscientific Model Development.

[CR134] Kim J-H, Park J-M, Lee S-B, Pudasainee D, Seo Y-C (2010). Anthropogenic mercury emission inventory with emission factors and total emission in Korea. Atmospheric Environment.

[CR133] Kim H, Soerensen AL, Hur J, Heimburger LE, Hahm D, Rhee TS, Noh S, Han S (2017). Methylmercury mass budgets and distribution characteristics in the Western Pacific Ocean. Environmental Science and Technology.

[CR135] Kirk JL, Muir DC, Antoniades D, Douglas MS, Evans MS, Jackson TA, Kling H, Lamoureux S (2011). Climate change and mercury accumulation in Canadian high and subarctic lakes. Environmental Science and Technology.

[CR136] Knightes CD, Sunderland EM, Craig Barber M, Johnston JM, Ambrose RB (2009). Application of ecosystem-scale fate and bioaccumulation models to predict fish mercury response times to changes in atmospheric deposition. Environmental Toxicology and Chemistry.

[CR900] Kocman, D., S.J. Wilson, H.M. Amos, K.H. Telmer, F. Steenhuisen, E.M. Sunderland, R.P. Mason, P. Outridge, et al. 2017. Toward an assesment of the global inventory of present-day mercury releases to freshwater environments. *International Journal of Environmental Research and Public Health* 14: 138. 10.3390/ijerph14020138 PMC5334692 28157152

[CR137] Kolka RK, Sturtevant BR, Miesel JR, Singh A, Wolter PT, Fraver S, DeSutter TM, Townsend PA (2017). Emissions of forest floor and mineral soil carbon, nitrogen and mercury pools and relationships with fire severity for the Pagami Creek Fire in the Boreal Forest of northern Minnesota. International Journal of Wildland Fire.

[CR138] Krabbenhoft DP, Sunderland EM (2013). Environmental science. Global change and mercury. Science.

[CR139] Kronberg R-M, Drott A, Jiskra M, Wiederhold JG, Björn E, Skyllberg U (2016). Forest harvest contribution to Boreal freshwater methyl mercury load. Global Biogeochemical Cycles.

[CR901] Kumar, A., S. Wu, Y. Huang, H. Liao, and J.O. Kaplan. 2017. Mercury from wildfires: Global emission inventories and sensitivity to 2000–2050 global change. *Atmospheric Environment* 173: 6–15.

[CR140] Kwon SY, Blum JD, Nadelhoffer KJ, Timothy Dvonch J, Tsui MT (2015). Isotopic study of mercury sources and transfer between a freshwater lake and adjacent forest food web. Science of the Total Environment.

[CR143] Lamborg CH, Hammerschmidt CR, Gill GA, Mason RP, Gichuki S (2012). An intercomparison of procedures for the determination of total mercury in seawater and recommendations regarding mercury speciation during GEOTRACES cruises. Limnology and Oceanography: Methods.

[CR142] Lamborg CH, Hammerschmidt CR, Bowman KL, Swarr GJ, Munson KM, Ohnemus DC, Lam PJ, Heimburger LE (2014). A global ocean inventory of anthropogenic mercury based on water column measurements. Nature.

[CR141] Lamborg CH, Hammerschmidt CR, Bowman KL (2016). An examination of the role of particles in oceanic mercury cycling. Philosophical Transactions. Series A, Mathematical, Physical, and Engineering Sciences.

[CR144] Landers DH, Simonich SL, Jaffe DA, Geiser LH, Campbell DH, Schwindt A, Schreck CB, Kent ML (2008). The fate, transport, and ecological impacts of airborne contaminants in western National Parks (USA).

[CR145] Lee X, Benoit G, Hu X (2000). Total gaseous mercury concentration and flux over a coastal saltmarsh vegetation in Connecticut, USA. Atmospheric Environment.

[CR146] Lehnherr I, St VL, Louis H Hintelmann, Kirk JL (2011). Methylation of inorganic mercury in polar marine waters. Nature Geoscience.

[CR147] Lei H, Wuebbles DJ, Liang XZ, Tao Z, Olsen S, Artz R, Ren X, Cohen M (2014). Projections of atmospheric mercury levels and their effect on air quality in the United States. Atmospheric Chemistry and Physics.

[CR148] Lepak RF, Yin R, Krabbenhoft DP, Ogorek JM, DeWild JF, Holsen TM, Hurley JP (2015). Use of Stable isotope signatures to determine mercury sources in the Great Lakes. Environmental Science & Technology Letters.

[CR149] Li M, Schartup AT, Valberg AP, Ewald JD, Krabbenhoft DP, Yin R, Balcom PH, Sunderland EM (2016). Environmental origins of methylmercury accumulated in subarctic estuarine fish indicated by mercury stable isotopes. Environmental Science and Technology.

[CR150] Lin C-J, Pongprueksa P, Lindberg SE, Pehkonen SO, Byun D, Jang C (2006). Scientific uncertainties in atmospheric mercury models I: Model science evaluation. Atmospheric Environment.

[CR152] Lindberg SE, Hanson PJ, Meyers TP, Kim KH (1998). Air/surface exchange of mercury vapor over forests: The need for a reassessment of continental biogenic emissions. Atmospheric Environment.

[CR151] Lindberg S, Bullock R, Ebinghaus R, Engstrom D, Feng X, Fitzgerald W, Pirrone N, Prestbo E (2007). A synthesis of progress and uncertainties in attributing the sources of mercury in deposition. Ambio.

[CR155] Lyman SN, Jaffe DA (2011). Formation and fate of oxidized mercury in the upper troposphere and lower stratosphere. Nature Geoscience.

[CR154] Lyman SN, Gustin MS, Prestbo EM, Kilner PI, Edgerton E, Hartsell B (2009). Testing and application of surrogate surfaces for understanding potential gaseous oxidized mercury dry deposition. Environmental Science and Technology.

[CR153] Lyman SN, Gustin MS, Prestbo EM (2010). A passive sampler for ambient gaseous oxidized mercury concentrations. Atmospheric Environment.

[CR156] Lyman SN, Jaffe DA, Gustin MS (2010). Release of mercury halides from KCl denuders in the presence of ozone. Atmospheric Chemistry and Physics.

[CR157] MacMillan GA, Girard C, Chetelat J, Laurion I, Amyot M (2015). High methylmercury in arctic and subarctic ponds is related to nutrient levels in the warming Eastern Canadian Arctic. Environmental Science and Technology.

[CR158] Mao H, Cheng I, Zhang L (2016). Current understanding of the driving mechanisms for spatiotemporal variations of atmospheric speciated mercury: A review. Atmospheric Chemistry and Physics.

[CR159] Martin LG, Labuschagne C, Brunke E-G, Weigelt A, Ebinghaus R, Slemr F (2017). Trend of atmospheric mercury concentrations at Cape Point for 1995–2004 and since 2007. Atmospheric Chemistry and Physics.

[CR160] Marusczak N, Sonke JE, Fu X, Jiskra M (2017). Tropospheric GOM at the Pic du Midi Observatory: Correcting bias in denuder based observations. Environmental Science and Technology.

[CR161] Masbou J, Point D, Sonke JE, Frappart F, Perrot V, Amouroux D, Richard P, Becker PR (2015). Hg stable isotope time trend in ringed seals registers decreasing sea ice cover in the Alaskan Arctic. Environmental Science and Technology.

[CR162] Masekoameng KE, Leaner J, Dabrowski J (2010). Trends in anthropogenic mercury emissions estimated for South Africa during 2000–2006. Atmospheric Environment.

[CR163] McClure CD, Jaffe DA, Edgerton ES (2014). Evaluation of the KCl denuder method for gaseous oxidized mercury using HgBr 2 at an in-service AMNet site. Environmental Science and Technology.

[CR164] McClure CD, Jaffe DA, Edgerton ES (2014). Evaluation of the KCl denuder method for gaseous oxidized mercury using HgBr2 at an in-service AMNet site. Environmental Science and Technology.

[CR165] Megaritis AG, Murphy BN, Racherla PN, Adams PJ, Pandis SN (2014). Impact of climate change on mercury concentrations and deposition in the eastern United States. Science of the Total Environment.

[CR166] Meili M (1991). The coupling of mercury and organic matter in the biogeochemical cycle: Towards a mechanistic model for the boreal forest zone. Water, Air, and Soil pollution.

[CR167] Melendez-Perez JJ, Fostier AH, Carvalho JA, Windmöller CC, Santos JC, Carpi A (2014). Soil and biomass mercury emissions during a prescribed fire in the Amazonian rain forest. Atmospheric Environment.

[CR168] Mitchell CP, Kolka RK, Fraver S (2012). Singular and combined effects of blowdown, salvage logging, and wildfire on forest floor and soil mercury pools. Environmental Science and Technology.

[CR169] Moore CW, Castro MS (2012). Investigation of factors affecting gaseous mercury concentrations in soils. Science of the Total Environment.

[CR170] Muir DC, Wang X, Yang F, Nguyen N, Jackson TA, Evans MS, Douglas M, Kock G (2009). Spatial trends and historical deposition of mercury in eastern and northern Canada inferred from lake sediment cores. Environmental Science and Technology.

[CR171] Munson KM, Lamborg CH, Swarr GJ, Saito MA (2015). Mercury species concentrations and fluxes in the Central Tropical Pacific Ocean. Global Biogeochemical Cycles.

[CR172] Muntean M, Janssens-Maenhout G, Song S, Selin NE, Olivier JG, Guizzardi D, Maas R, Dentener F (2014). Trend analysis from 1970 to 2008 and model evaluation of EDGARv4 global gridded anthropogenic mercury emissions. Science of the Total Environment.

[CR173] Nansai K, Oguchi M, Suzuki N, Kida A, Nataami T, Tanaka C, Haga M (2012). High-resolution inventory of Japanese anthropogenic mercury emissions. Environmental Science and Technology.

[CR174] Navrátil T, Shanley J, Rohovec J, Hojdová M, Penížek V, Buchtová J (2014). Distribution and pools of mercury in Czech forest soils. Water, Air, and Soil pollution.

[CR175] Navrátil T, Shanley JB, Rohovec J, Oulehle F, Šimeček M, Houška J, Cudlín P (2016). Soil mercury distribution in adjacent coniferous and deciduous stands highly impacted by acid rain in the Ore Mountains, Czech Republic. Applied Geochemistry.

[CR176] Ndu U, Barkay T, Mason RP, Traore Schartup A, Al-Farawati R, Liu J, Reinfelder JR (2015). The use of a mercury biosensor to evaluate the bioavailability of mercury-thiol complexes and mechanisms of mercury uptake in bacteria. PLoS ONE.

[CR177] Nelson PF, Morrison AL, Malfroy HJ, Cope M, Lee S, Hibberd ML, Meyer CP, McGregor J (2012). Atmospheric mercury emissions in Australia from anthropogenic, natural and recycled sources. Atmospheric Environment.

[CR178] Nerentorp Mastromonaco M, Gårdfeldt K, Jourdain B, Abrahamsson K, Granfors A, Ahnoff M, Dommergue A, Méjean G (2016). Antarctic winter mercury and ozone depletion events over sea ice. Atmospheric Environment.

[CR179] Obrist D (2007). Atmospheric mercury pollution due to losses of terrestrial carbon pools?. Biogeochemistry.

[CR180] Obrist D (2012). Mercury distribution across 14 U.S. forests. Part II: Patterns of methyl mercury concentrations and areal mass of total and methyl mercury. Environmental Science and Technology.

[CR182] Obrist D, Gustin MS, Arnone JA, Johnson DW, Schorran DE, Verburg PSJ (2005). Measurements of gaseous elemental mercury fluxes over intact tallgrass prairie monoliths during one full year. Atmospheric Environment.

[CR184] Obrist D, Johnson DW, Lindberg SE, Luo Y, Hararuk O, Bracho R, Battles JJ, Dail DB (2011). Mercury distribution across 14 U.S. Forests. Part I: Spatial patterns of concentrations in biomass, litter, and soils. Environmental Science and Technology.

[CR183] Obrist D, Johnson DW, Edmonds RL (2012). Effects of vegetation type on mercury concentrations and pools in two adjacent coniferous and deciduous forests. Journal of Plant Nutrition and Soil Science.

[CR186] Obrist D, Pokharel AK, Moore C (2014). Vertical profile measurements of soil air suggest immobilization of gaseous elemental mercury in mineral soil. Environmental Science and Technology.

[CR185] Obrist D, Pearson C, Webster J, Kane T, Lin CJ, Aiken GR, Alpers CN (2016). A synthesis of terrestrial mercury in the western United States: Spatial distribution defined by land cover and plant productivity. Science of the Total Environment.

[CR181] Obrist D, Agnan Y, Jiskra M, Olson CL, Colegrove DP, Hueber J, Moore CW, Sonke JE (2017). Tundra uptake of atmospheric elemental mercury drives Arctic mercury pollution. Nature.

[CR187] Ortiz VL, Mason RP, Ward JE (2015). An examination of the factors influencing mercury and methylmercury particulate distributions, methylation and demethylation rates in laboratory-generated marine snow. Marine Chemistry.

[CR188] Outridge PM, Rausch N, Percival JB, Shotyk W, McNeely R (2011). Comparison of mercury and zinc profiles in peat and lake sediment archives with historical changes in emissions from the Flin Flon metal smelter, Manitoba, Canada. Science of the Total Environment.

[CR189] Pacyna EG, Pacyna JM, Sundseth K, Munthe J, Kindbom K, Wilson S, Steenhuisen F, Maxson P (2010). Global emission of mercury to the atmosphere from anthropogenic sources in 2005 and projections to 2020. Atmospheric Environment.

[CR190] Pacyna JM, Travnikov O, De Simone F, Hedgecock IM, Sundseth K, Pacyna EG, Steenhuisen F, Pirrone N (2016). Current and future levels of mercury atmospheric pollution on a global scale. Atmospheric Chemistry and Physics.

[CR191] Parks JM, Johs A, Podar M, Bridou R, Hurt RA, Smith SD, Tomanicek SJ, Qian Y (2013). The genetic basis for bacterial mercury methylation. Science.

[CR192] Peña-Rodríguez S, Pontevedra-Pombal X, Fernández-Calviño D, Taboada T, Arias-Estévez M, Martínez-Cortizas A, Nóvoa-Muñoz JC, García-Rodeja E (2012). Mercury content in volcanic soils across Europe and its relationship with soil properties. Journal of Soils and Sediments.

[CR193] Peña-Rodríguez S, Pontevedra-Pombal X, Gayoso EG-R, Moretto A, Mansilla R, Cutillas-Barreiro L, Arias-Estévez M, Nóvoa-Muñoz JC (2014). Mercury distribution in a toposequence of sub-Antarctic forest soils of Tierra del Fuego (Argentina) as consequence of the prevailing soil processes. Geoderma.

[CR194] Perry E, Norton SA, Kamman NC, Lorey PM, Driscoll CT (2005). Deconstruction of historic mercury accumulation in lake sediments, northeastern United States. Ecotoxicology.

[CR196] Pirrone N, Cinnirella S, Feng X, Finkelman RB, Friedli HR, Leaner J, Mason R, Mukherjee AB (2010). Global mercury emissions to the atmosphere from anthropogenic and natural sources. Atmospheric Chemistry and Physics.

[CR195] Pirrone N, Aas W, Cinnirella S, Ebinghaus R, Hedgecock IM, Pacyna J, Sprovieri F, Sunderland EM (2013). Toward the next generation of air quality monitoring: Mercury. Atmospheric Environment.

[CR197] Podar M, Gilmour CC, Brandt CC, Soren A, Brown SD, Crable BR, Palumbo AV, Somenahally AC (2015). Global prevalence and distribution of genes and microorganisms involved in mercury methylation. Science Advances.

[CR198] Point D, Sonke JE, Day RD, Roseneau DG, Hobson KA, Vander Pol SS, Moors AJ, Pugh RS (2011). Methylmercury photodegradation influenced by sea-ice cover in Arctic marine ecosystems. Nature Geoscience.

[CR199] Prestbo EM, Gay DA (2009). Wet deposition of mercury in the U.S. and Canada, 1996–2005: Results and analysis of the NADP mercury deposition network (MDN). Atmospheric Environment.

[CR200] Rafaj P, Bertok I, Cofala J, Schöpp W (2013). Scenarios of global mercury emissions from anthropogenic sources. Atmospheric Environment.

[CR201] Richardson JB, Friedland AJ (2015). Mercury in coniferous and deciduous upland forests in Northern New England, USA: Implications from climate change. Biogeosciences Discussions.

[CR202] Richardson JB, Friedland AJ, Engerbretson TR, Kaste JM, Jackson BP (2013). Spatial and vertical distribution of mercury in upland forest soils across the northeastern United States. Environmental Pollution.

[CR203] Risch MR, Dewild JF, Krabbenhoft DP, Kolka RK, Zhang L (2012). Litterfall mercury dry deposition in the eastern USA. Environmental Pollution.

[CR204] Risch MR, Gay DA, Fowler KK, Keeler GJ, Backus SM, Blanchard P, Barres JA, Dvonch JT (2012). Spatial patterns and temporal trends in mercury concentrations, precipitation depths, and mercury wet deposition in the North American Great Lakes region, 2002–2008. Environmental Pollution.

[CR205] Rydberg J, Rosch M, Heinz E, Biester H (2015). Influence of catchment vegetation on mercury accumulation in lake sediments from a long-term perspective. Science of the Total Environment.

[CR206] Schaefer JK, Kronberg RM, Morel FM, Skyllberg U (2014). Detection of a key Hg methylation gene, hgcA, in wetland soils. Environmental Microbiology Reports.

[CR207] Schartup AT, Balcom PH, Soerensen AL, Gosnell KJ, Calder RS, Mason RP, Sunderland EM (2015). Freshwater discharges drive high levels of methylmercury in Arctic marine biota. Proceedings of the National Academy of Sciences United States of America.

[CR208] Schauble EA (2007). Role of nuclear volume in driving equilibrium stable isotope fractionation of mercury, thallium, and other very heavy elements. Geochimica et Cosmochimica Acta.

[CR209] Schwesig D, Matzner E (2000). Pools and fluxes of mercury and methylmercury in two forested catchments in Germany. Science of the Total Environment.

[CR211] Selin NE (2014). Global change and mercury cycling: Challenges for implementing a global mercury treaty. Environmental Toxicology and Chemistry.

[CR212] Selin NE, Jacob DJ, Park RJ, Yantosca RM, Strode S, Jaegle L, Jaffe D (2007). Chemical cycling and deposition of atmospheric mercury: Global constraints from observations. Journal of Geophysical Research.

[CR213] Selin NE, Jacob DJ, Yantosca RM, Strode S, Jaeglé L, Sunderland EM (2008). Global 3-D land-ocean-atmosphere model for mercury: Present-day versus preindustrial cycles and anthropogenic enrichment factors for deposition. Global Biogeochemical Cycles.

[CR210] Selin, H., S.E. Keane, S. Wang, N.E. Selin, K. Davis, and D. Bally. 2018. Linking science and policy to support the implementation of the Minamata Convention on Mercury. *Ambio*. 10.1007/s13280-017-1003-x. 10.1007/s13280-017-1003-x PMC5794682 29388129

[CR214] Semeniuk K, Dastoor A (2017). Development of a global ocean mercury model with a methylation cycle: Outstanding issues. Global Biogeochemical Cycles.

[CR215] Senn DB, Chesney EJ, Blum JD, Bank MS, Maage A, Shine JP (2010). Stable isotope (N, C, Hg) study of methylmercury sources and trophic transfer in the northern gulf of Mexico. Environmental Science and Technology.

[CR216] Shah V, Jaeglé L, Gratz LE, Ambrose JL, Jaffe DA, Selin NE, Song S, Campos TL (2016). Origin of oxidized mercury in the summertime free troposphere over the southeastern US. Atmospheric Chemistry and Physics.

[CR217] Sherman LS, Blum JD (2013). Mercury stable isotopes in sediments and largemouth bass from Florida lakes, USA. Science of the Total Environment.

[CR218] Sherman LS, Blum JD, Johnson KP, Keeler GJ, Barres JA, Douglas TA (2010). Mass-independent fractionation of mercury isotopes in Arctic snow driven by sunlight. Nature Geoscience.

[CR219] Sherman LS, Blum JD, Keeler GJ, Demers JD, Dvonch JT (2012). Investigation of local mercury deposition from a coal-fired power plant using mercury isotopes. Environmental Science and Technology.

[CR220] Sigler JM, Lee X (2006). Gaseous mercury in background forest soil in the northeastern United States. Journal of Geophysical Research: Biogeosciences.

[CR222] Slemr F, Brunke E-G, Ebinghaus R, Temme C, Munthe J, Wängberg I, Schroeder W, Steffen A (2003). Worldwide trend of atmospheric mercury since 1977. Geophysical Research Letters.

[CR224] Slemr F, Brunke EG, Labuschagne C, Ebinghaus R (2008). Total gaseous mercury concentrations at the Cape Point GAW station and their seasonality. Geophysical Research Letters.

[CR223] Slemr F, Brunke EG, Ebinghaus R, Kuss J (2011). Worldwide trend of atmospheric mercury since 1995. Atmospheric Chemistry and Physics.

[CR225] Slemr F, Weigelt A, Ebinghaus R, Brenninkmeijer C, Baker A, Schuck T, Rauthe-Schöch A, Riede H (2014). Mercury plumes in the global upper troposphere observed during flights with the CARIBIC observatory from May 2005 until June 2013. Atmosphere.

[CR221] Slemr F, Brenninkmeijer CA, Rauthe-Schöch A, Weigelt A, Ebinghaus R, Brunke E-G, Martin L, Spain TG (2016). El Niño-Southern Oscillation influence on tropospheric mercury concentrations. Geophysical Research Letters.

[CR226] Smith-Downey NV, Sunderland EM, Jacob DJ (2010). Anthropogenic impacts on global storage and emissions of mercury from terrestrial soils: Insights from a new global model. Journal of Geophysical Research.

[CR231] Soerensen AL, Sunderland EM, Holmes CD, Jacob DJ, Yantosca RM, Skov H, Christensen JH, Strode SA (2010). An improved global model for air-sea exchange of mercury: High concentrations over the North Atlantic. Environmental Science and Technology.

[CR228] Soerensen AL, Jacob DJ, Streets DG, Witt MLI, Ebinghaus R, Mason RP, Andersson M, Sunderland EM (2012). Multi-decadal decline of mercury in the North Atlantic atmosphere explained by changing subsurface seawater concentrations. Geophysical Research Letters.

[CR230] Soerensen AL, Mason RP, Balcom PH, Sunderland EM (2013). Drivers of surface ocean mercury concentrations and air-sea exchange in the West Atlantic Ocean. Environmental Science and Technology.

[CR229] Soerensen AL, Mason RP, Balcom PH, Jacob DJ, Zhang Y, Kuss J, Sunderland EM (2014). Elemental mercury concentrations and fluxes in the tropical atmosphere and ocean. Environmental Science and Technology.

[CR227] Soerensen AL, Jacob DJ, Schartup AT, Fisher JA, Lehnherr I, St. Louis VL, Heimbürger L-E, Sonke JE (2016). A mass budget for mercury and methylmercury in the Arctic Ocean. Global Biogeochemical Cycles.

[CR232] Sommar J, Zhu W, Shang L, Lin C-J, Feng X (2016). Seasonal variations in metallic mercury (Hg^0^) vapor exchange over biannual wheat-corn rotation cropland in the North China Plain. Biogeosciences.

[CR234] Song S, Selin NE, Soerensen AL, Angot H, Artz R, Brooks S, Brunke EG, Conley G (2015). Top-down constraints on atmospheric mercury emissions and implications for global biogeochemical cycling. Atmospheric Chemistry and Physics.

[CR233] Song S, Selin NE, LE Gratz, Ambrose JL, Jaffe DA, Shah V, Jaeglé L, Giang A (2016). Constraints from observations and modeling on atmosphere–surface exchange of mercury in eastern North America. Elementa: Science of the Anthropocene.

[CR235] Sonke JE (2011). A global model of mass independent mercury stable isotope fractionation. Geochimica et Cosmochimica Acta.

[CR236] Sonke JE, Schäfer J, Chmeleff J, Audry S, Blanc G, Dupré B (2010). Sedimentary mercury stable isotope records of atmospheric and riverine pollution from two major European heavy metal refineries. Chemical Geology.

[CR239] Sprovieri F, Pirrone N, Ebinghaus R, Kock H, Dommergue A (2010). A review of worldwide atmospheric mercury measurements. Atmospheric Chemistry and Physics.

[CR238] Sprovieri F, Pirrone N, Bencardino M, D’Amore F, Carbone F, Cinnirella S, Mannarino V, Landis M (2016). Atmospheric mercury concentrations observed at ground-based monitoring sites globally distributed in the framework of the GMOS network. Atmospheric Chemistry and Physics.

[CR237] Sprovieri F, Pirrone N, Bencardino M, D’Amore F, Angot H, Barbante C, Brunke E-G, Arcega-Cabrera F (2017). Five-year records of mercury wet deposition flux at GMOS sites in the Northern and Southern hemispheres. Atmospheric Chemistry and Physics.

[CR240] St. Louis, V., J. Graydon, C. Mitchell, and C. Oswald. 2016. Mercury fate and methylation in terrestrial upland and wetland environments In Canada Mercury Science Assessment.

[CR241] Stern GA, Macdonald RW, Outridge PM, Wilson S, Chetelat J, Cole A, Hintelmann H, Loseto LL (2012). How does climate change influence Arctic mercury?. Science of the Total Environment.

[CR242] Streets D, Hao J, Wu Y, Jiang J, Chan M, Tian H, Feng X (2005). Anthropogenic mercury emissions in China. Atmospheric Environment.

[CR245] Streets DG, Zhang Q, Wu Y (2009). Projections of global mercury emissions in 2050. Environmental Science and Technology.

[CR243] Streets DG, Devane MK, Lu Z, Bond TC, Sunderland EM, Jacob DJ (2011). All-time releases of mercury to the atmosphere from human activities. Environmental Science and Technology.

[CR244] Streets DG, Horowitz HM, Jacob DJ, Lu Z, Levin L, Ter Schure AFH, Sunderland EM (2017). Total mercury released to the environment by human activities. Environmental Science and Technology.

[CR246] Strode SA, Jaeglé L, Selin NE, Jacob DJ, Park RJ, Yantosca RM, Mason RP, Slemr F (2007). Air-sea exchange in the global mercury cycle. Global Biogeochemical Cycles.

[CR247] Subir M, Ariya PA, Dastoor AP (2011). A review of uncertainties in atmospheric modeling of mercury chemistry I. Uncertainties in existing kinetic parameters: Fundamental limitations and the importance of heterogeneous chemistry. Atmospheric Environment.

[CR248] Sun G, Sommar J, Feng X, Lin CJ, Ge M, Wang W, Yin R, Fu X (2016). Mass-dependent and -independent fractionation of mercury isotope during gas-phase oxidation of elemental mercury vapor by atomic Cl and Br. Environmental Science and Technology.

[CR249] Sun R, Streets DG, Horowitz HM, Amos HM, Liu G, Perrot V, Toutain J-P, Hintelmann H (2016). Historical (1850–2010) mercury stable isotope inventory from anthropogenic sources to the atmosphere. Elementa: Science of the Anthropocene.

[CR250] Sunderland EM (2007). Mercury exposure from domestic and imported estuarine and marine fish in the U.S. seafood market. Environmental Health Perspectives.

[CR253] Sunderland EM, Mason RP (2007). Human impacts on open ocean mercury concentrations. Global Biogeochemical Cycles.

[CR252] Sunderland EM, Krabbenhoft DP, Moreau JW, Strode SA, Landing WM (2009). Mercury sources, distribution, and bioavailability in the North Pacific Ocean: Insights from data and models. Global Biogeochemical Cycles.

[CR251] Sunderland EM, Dalziel J, Heyes A, Branfireun BA, Krabbenhoft DP, Gobas FAPC (2010). Response of a macrotidal estuary to changes in anthropogenic mercury loading between 1850 and 2000. Environmental Science and Technology.

[CR254] Temme C, Blanchard P, Steffen A, Banic C, Beauchamp S, Poissant L, Tordon R, Wiens B (2007). Trend, seasonal and multivariate analysis study of total gaseous mercury data from the Canadian atmospheric mercury measurement network (CAMNet). Atmospheric Environment.

[CR255] Tørseth K, Aas W, Breivik K, Fjæraa AM, Fiebig M, Hjellbrekke AG, Lund Myhre C, Solberg S (2012). Introduction to the European Monitoring and Evaluation Programme (EMEP) and observed atmospheric composition change during 1972–2009. Atmospheric Chemistry and Physics.

[CR256] Townsend JM, Driscoll CT, Rimmer CC, McFarland KP (2014). Avian, salamander, and forest floor mercury concentrations increase with elevation in a terrestrial ecosystem. Environmental Toxicology and Chemistry.

[CR258] Travnikov O, Ilyin I, Pirrone N, Mason RP (2009). The EMEP/MSC-E Mercury Modeling System. Mercury fate and transport in the global atmosphere: Emissions, measurements, and models.

[CR257] Travnikov O, Angot H, Artaxo P, Bencardino M, Bieser J, D’Amore F, Dastoor A, De Simone F (2017). Multi-model study of mercury dispersion in the atmosphere: Atmospheric processes and model evaluation. Atmospheric Chemistry and Physics.

[CR259] Tsui MT, Blum JD, Kwon SY, Finlay JC, Balogh SJ, Nollet YH (2012). Sources and transfers of methylmercury in adjacent river and forest food webs. Environmental Science and Technology.

[CR260] Ullrich SM, Tanton TW, Abdrashitova SA (2001). Mercury in the aquatic environment: A review of factors affecting methylation. Critical Reviews in Environmental Science and Technology.

[CR261] UNEP (2013). Global Mercury Assessment 2013: Sources, Emissions, Releases and Environmental Transport.

[CR262] United Nations Environment Programme (2013). Global mercury assessment 2013: Sources, emissions, releases, and environmental transport.

[CR263] US EPA. 2015. 2011 National Emissions Inventory (version 2) Technical Support Document. United States Environmental Protection Agency, Research Triangle Park, NC.

[CR265] Wang SX, Song JX, Li GH, Wu Y, Zhang L, Wan Q, Streets DG, Chin CK (2010). Estimating mercury emissions from a zinc smelter in relation to China’s mercury control policies. Environmental Pollution.

[CR264] Wang F, Saiz-Lopez A, Mahajan AS, Gómez Martín JC, Armstrong D, Lemes M, Hay T, Prados-Roman C (2014). Enhanced production of oxidised mercury over the tropical Pacific Ocean: A key missing oxidation pathway. Atmospheric Chemistry and Physics.

[CR266] Wang X, Bao Z, Lin C-J, Yuan W, Feng X (2016). Assessment of global mercury deposition through litterfall. Environmental Science and Technology.

[CR267] Wang X, Luo J, Yin R, Yuan W, Lin CJ, Sommar J, Feng X, Wang H (2017). Using mercury isotopes to understand mercury accumulation in the montane forest floor of the Eastern Tibetan Plateau. Environmental Science and Technology.

[CR268] Webster JP, Kane TJ, Obrist D, Ryan JN, Aiken GR (2016). Estimating mercury emissions resulting from wildfire in forests of the Western United States. Science of the Total Environment.

[CR269] Weigelt A, Ebinghaus R, Manning AJ, Derwent RG, Simmonds PG, Spain TG, Jennings SG, Slemr F (2015). Analysis and interpretation of 18 years of mercury observations since 1996 at Mace Head, Ireland. Atmospheric Environment.

[CR270] Weiss-Penzias PS, Gay DA, Brigham ME, Parsons MT, Gustin MS, Ter Schure A (2016). Trends in mercury wet deposition and mercury air concentrations across the U.S. and Canada. Science of the Total Environment.

[CR271] Westerling AL, Hidalgo HG, Cayan DR, Swetnam TW (2006). Warming and earlier spring increase western U.S. forest wildfire activity. Science.

[CR272] Wiederhold JG, Cramer CJ, Daniel K, Infante I, Bourdon B, Kretzschmar R (2010). Equilibrium mercury isotope fractionation between dissolved Hg(II) species and thiol-bound Hg. Environmental Science and Technology.

[CR273] Wiedinmyer C, Friedli H (2007). Mercury emission estimates from fires: An initial inventory for the United States. Environmental Science and Technology.

[CR274] Woodruff LG, Cannon WF (2010). Immediate and long-term fire effects on total mercury in forests soils of northeastern Minnesota. Environmental Science and Technology.

[CR275] Wright LP, Zhang L, Marsik FJ (2016). Overview of mercury dry deposition, litterfall, and throughfall studies. Atmospheric Chemistry and Physics.

[CR276] Yang Y, Yanaia RD, Montesdeoca M, Driscoll CT (2017). Measuring mercury in wood: Challenging but important. International Journal of Environmental Analytical Chemistry.

[CR277] Ye Z, Mao H, Lin C-J, Kim SY (2016). Investigation of processes controlling summertime gaseous elemental mercury oxidation at midlatitudinal marine, coastal, and inland sites. Atmospheric Chemistry and Physics.

[CR279] Yu RQ, Reinfelder JR, Hines ME, Barkay T (2013). Mercury methylation by the methanogen *Methanospirillum hungatei*. Applied and Environmental Microbiology.

[CR278] Yu B, Fu X, Yin R, Zhang H, Wang X, Lin C-J, Wu C, Zhang Y (2016). Isotopic composition of atmospheric mercury in China: New evidence for source and transformation processes in air and in vegetation. Environmental Science and Technology.

[CR289] Zhang Y, Jaeglé L (2013). Decreases in mercury wet deposition over the United States during 2004–2010: Roles of domestic and global background emission reductions. Atmosphere.

[CR285] Zhang L, Wright LP, Blanchard P (2009). A review of current knowledge concerning dry deposition of atmospheric mercury. Atmospheric Environment.

[CR283] Zhang L, Wang S, Wu Q, Meng Y, Yang H, Wang F, Hao J (2012). Were mercury emission factors for Chinese non-ferrous metal smelters overestimated? Evidence from onsite measurements in six smelters. Environmental Pollution.

[CR281] Zhang H, Yin RS, Feng XB, Sommar J, Anderson CW, Sapkota A, Fu XW, Larssen T (2013). Atmospheric mercury inputs in montane soils increase with elevation: Evidence from mercury isotope signatures. Science Reports.

[CR290] Zhang Y, Jaeglé L, Thompson L (2014). Natural biogeochemical cycle of mercury in a global three-dimensional ocean tracer model. Global Biogeochemical Cycles.

[CR282] Zhang L, Wang S, Wang L, Wu Y, Duan L, Wu Q, Wang F, Yang M (2015). Updated emission inventories for speciated atmospheric mercury from anthropogenic sources in China. Environmental Science and Technology.

[CR287] Zhang Y, Jacob DJ, Dutkiewicz S, Amos HM, Long MS, Sunderland EM (2015). Biogeochemical drivers of the fate of riverine mercury discharged to the global and Arctic oceans. Global Biogeochemical Cycles.

[CR280] Zhang H, Holmes CD, Wu S (2016). Impacts of changes in climate, land use and land cover on atmospheric mercury. Atmospheric Environment.

[CR284] Zhang L, Wang S, Wu Q, Wang F, Lin C-J, Zhang L, Hui M, Yang M (2016). Mercury transformation and speciation in flue gases from anthropogenic emission sources: A critical review. Atmospheric Chemistry and Physics.

[CR286] Zhang L, Wu Z, Cheng I, Wright LP, Olson ML, Gay DA, Risch MR, Brooks S (2016). The estimated six-year mercury dry deposition across North America. Environmental Science and Technology.

[CR288] Zhang Y, Jacob DJ, Horowitz HM, Chen L, Amos HM, Krabbenhoft DP, Slemr F, St Louis VL (2016). Observed decrease in atmospheric mercury explained by global decline in anthropogenic emissions. Proceedings of the National Academy of Sciences of the United States of America.

[CR291] Zheng W, Obrist D, Weis D, Bergquist BA (2016). Mercury isotope compositions across North American forests. Global Biogeochemical Cycles.

[CR292] Zhu W, Lin C-J, Wang X, Sommar J, Fu X, Feng X (2016). Global observations and modeling of atmosphere–surface exchange of elemental mercury: A critical review. Atmospheric Chemistry and Physics.

